# An experiment on deception, reputation and trust

**DOI:** 10.1007/s10683-020-09681-9

**Published:** 2020-12-01

**Authors:** David Ettinger, Philippe Jehiel

**Affiliations:** 1 Université Paris-Dauphine, PSL Research University, CNRS, IRD, LEDa, 75016 Paris, France; 2 PSE, 48 Boulevard Jourdan, 75014 Paris, France; 3 University College London, London, UK

**Keywords:** C72, D82

## Abstract

An experiment is designed to shed light on how deception works. The experiment involves a twenty period sender/receiver game in which period 5 has more weight than other periods. In each period, the informed sender communicates about the realized state, the receiver then reports a belief about the state before being informed whether the sender lied. Throughout the interaction, a receiver is matched with the same sender who is either malevolent with an objective opposed to the receiver or benevolent always telling the truth. The main findings are: (1) in several variants (differing in the weight of the key period and the share of benevolent senders), the deceptive tactic in which malevolent senders tell the truth up to the key period and then lie at the key period is used roughly 25% of the time, (2) the deceptive tactic brings higher expected payoff than other observed strategies, and (3) a majority of receivers do not show cautiousness at the key period when no lie was made before. These observations do not match the predictions of the Sequential Equilibrium and can be organized using the analogy-based sequential equilibrium (ABSE) in which three quarters of subjects reason coarsely.

**Electronic supplementary material:**

The online version of this article (10.1007/s10683-020-09681-9) contains supplementary material, which is available to authorized users.

## Introduction

During World War II, the Red Orchestra was the most important spying network of the Soviet Union. It was used to send information to Moscow through radio transmissions. The Germans managed to get control over the Red Orchestra network, and to convince some of its members to work for them. Then, began a new strategy: the Funkspiel. Rather than interrupting the Red Orchestra transmissions, the Germans kept on using it to send information to Moscow. Not only did they send information, but, at least initially, they even sent accurate and important pieces of information. One can guess that the idea of the Germans was to maintain a high level of trust in the mind of the Russians regarding the quality of the Red Orchestra information (because Moscow also knew that radio transmitters could be detected), and to use this communication network to intoxicate the Russian services at a key moment.^1^

The Red Orchestra can be viewed as providing an illustration of the kind of deceptive tactics this paper is about. Cialdini (2006) in his best-seller book on influence provides another illustration. At the end of the chapter on authority, Cialdini reports the story of a waiter named Vincent who was particularly successful at increasing the bill and the tip that goes with it in large party dinners. After letting the first customer in the party make her choice of starter, Vincent would invariably suggest that the pick was not the best on that evening, and would redirect the customer on a cheaper starter. By gaining the trust of the customers, Vincent was able to put forward his recommendation of the most expensive wine afterwards, which would increase considerably the bill.

The cases of the Red Orchestra and of the waiter Vincent are vivid examples of repeated information transmission situations in which the agent/organization sending the information and the agent/organization receiving the information may possibly have conflicting interests, and some pieces of information may be attached to higher stakes. We believe that there are many environments with similar characteristics. To suggest a very different context, consider the everyday life of politicians: they intervene frequently in the media and elsewhere (the communication aspect); they sometimes care more about being reelected than just telling the truth about the state of the economy (the potential difference of objective between the sender and the receiver), and as reelection time approaches, they become more anxious to convey the belief that they are highly competent and trustworthy (the high stake event).^2^

The key strategic considerations in such multi-stage information transmission environments are: (1) How does the receiver use the information he gets to assess the likelihood of the current state of affairs but also to assess the type of sender he is facing (which may be useful to interpret subsequent messages)? (2) How does the sender understand and make use of the receiver’s inference process? (3) On the receiver side, it requires understanding how trust or credibility evolves. (4) On the sender side, it requires understanding the extent to which deception or other manipulative tactics are effective.

This paper proposes an experimental approach to shed light on deception, reputation, credibility, and trust. Specifically, we summarize below results found in experiments on a repeated information transmission game (à la Sobel 1985) in which the stake of one period is much higher than that of other periods in agreement with the motivating examples discussed above. Senders and receivers are randomly matched at the start of the interaction. Each sender/receiver pair plays the same stage game during twenty periods. In each period, a new state is drawn at random, the sender is perfectly informed of the state of the world while the receiver is not. The sender sends a message regarding the state of the world, then the receiver chooses an action with the objective of matching the true state. The sender is either benevolent and always sends a truthful message (a truthtelling machine in the experiment) or she is malevolent in which case her objective is to induce actions of the receiver as far as possible from the true states of the world. The receiver does not know the type of the sender he is matched with, but he discovers at the end of each period whether the message received during this period was truthful or not. Furthermore, the 5th period is the high stake period, having much more impact than other periods on the overall payoff of both the sender and the receiver (in the Red Orchestra, this high stake could correspond to a key offensive).

In our baseline treatment, the key period contributes five times as much as the other periods to the overall payoff (we say the weight of this period is five), and the initial share of benevolent senders represents 20% of all senders. In this treatment, receivers were not informed that human senders had preferences opposed to them, and they were only told that such senders lie on average half of the time.^3^

We considered several variants of the baseline treatment either increasing the weight of the key period to 10 or reducing the share of benevolent senders to 10% or letting the receivers know about the preferences of human senders. We obtained the following results:

- In the baseline treatment, a large share of senders (roughly 28%), chooses the following deceptive tactic: they send truthful messages up to the period just before the key period and then send a false message in the key period. The share of deceptive tactics followed by malevolent senders is roughly the same whether the initial proportion of benevolent senders is 10% or 20% and whether the weight of the key period is 5 or 10.
- Receivers are (in aggregate) deceived by this strategy. In the key period, they trust too much a sender who has only sent truthful messages until the key period (i.e., they choose an action which is too close to the message they receive as compared to what would be optimal to do). The deceptive tactic is successful.
- The behaviors are roughly the same whether or not receivers are informed of senders’ preferences but, this is true when subjects play the game for the first two times, while some learning effect is observed after more plays of the game.

Assuming subjects behave as in the sequential equilibrium (SE) of the game does not provide a good account of the observations for several reasons: (1) Senders follow the deceptive tactic too often. (2) The deceptive tactic is successful in the sense that, in our data, deceptive senders obtain higher payoffs than non-deceptive senders while sequential equilibrium would predict that all employed strategies should be equally good. (3) While the sequential equilibrium would predict that the share of senders following the deceptive tactic should increase if the weight of the key period increases and/or if the initial proportion of benevolent senders increases, we see no such comparative statics in our data.

Faced with these observations, we suggest interpreting our findings by considering that at least some share of our subjects followed an inference process that is less sophisticated than the one involved in SE. Specifically, given that receivers knew that human senders lie overall half of the time, a simple (though naive) inference process for coarse receivers consists in believing that human senders lie half of the time in every period independently of the history (with a corresponding Bayesian updating process as no lies are observed). If a human sender knew she was facing such a coarse receiver and given her preferences, she would pick the deceptive tactic. Indeed, by telling the truth up to the period just before the key period, she would increase considerably the belief in the coarse receiver’s mind that she is a benevolent machine, which she could exploit at the key period by lying (similarly as in the waiter Vincent story).

The kind of reasoning just proposed involving naive inference on the receiver side and deception on the sender side -while at odds with SE- can be captured in the framework of the analogy-based sequential equilibrium (ABSE) developed in Ettinger and Jehiel (2010) (see Jehiel (2005) for the exposition of the analogy-based expectation equilibrium in complete information settings on which EJ build and Jehiel and Samuelson (2012) for an application of ABSE). We observe within the ABSE framework that allowing subjects -senders or receivers- to be either coarse^4^ with probability 3/4 or rational with probability 1/4 provides a good account of the (qualitative) observations made above.

Our study relates to different strands of experimental literature. First, it relates to the experimental literature on reputation in games as initiated by Camerer and Weigelt (1988), Neral and Ochs (1992) and Jung et al. (1994) which considers reputation games such as the chain-store game or the borrower-lender game. A key difference with that literature is our focus on repeated sender/receiver communication games in which there is no value for a malevolent sender to being permanently confounded with a machine always telling the truth, but only a value to being temporarily confounded so as to take advantage of it in the key period.^5^ Interestingly, previous studies on reputation games have suggested that the sequential equilibrium may be a good tool to organize the data,^6^ which contrasts with our finding that theories beyond the sequential equilibrium are needed to give a reasonable account of the data in our experiment.

Our study is also related to a lesser extent to the experimental literature on non-repeated strategic information transmission games à la Crawford and Sobel (1982) that was initiated by Dickhaut et al. (1995) and Blume et al. (1998) (see also Blume et al. (2001), Cai and Wang (2006), Kawagoe and Takizawa (2009) or Wang et al. (2010)). That literature has noted that senders have a tendency to transmit more information than theory predicts suggesting that (at least some) senders may be averse to lying.^7^ It has also suggested that receivers may be more credulous than theory predicts. Our study is complementary to that strand of literature to the extent that our main interest is focused on the timing of the lies and the dynamic inference process which cannot be studied in non-repeated communication games.

## The game and some theoretical benchmarks

We consider a game played by an informed sender and an uninformed receiver. The game consists of twenty periods. At the beginning of each period *k*, the sender (but not the receiver) is informed of the state of the world $$ s_{k}$$ prevailing in this period. The receiver discovers $$s_{k}$$ at the end of period *k*. States of the world may take two values, 0 and 1. The states of the world in the different periods are independently drawn with a probability $$\frac{1}{2}$$ for each realization.

In each period *k*, the sender sends a message $$m_{k}$$ which can be equal to 0 or 1: $$m_{k}$$ is supposed to be representing the current state of the world. The sender can choose a truthful ($$m_{k}=s_{k}$$) or a false ($$ m_{k}=1-s_{k}$$) message about the state of the world. The receiver observes the message $$m_{k}$$, but does not observe whether the message is truthful or false (the receiver is aware that the sender may choose strategically to send a false message). Then, the receiver makes a decision $$a_{k}\in [0,1]$$ after which he is informed of $$s_{k}$$.

The receiver’s payoff in period *k* is equal to $\delta _{k}(1-(a_{k}-s_{k})^{2})$ where $\delta _{k}$ is the weight of period *k*. The overall payoff of the receiver is $\sum \nolimits _{k=1}^{20}\delta _{k}(1-(a_{k}-s_{k})^{2})$. The choice of a quadratic scoring rule ensures that if the receiver only considers the current period’s payoff, he will pick the action that corresponds to what he subjectively believes to be the expected value of $s_{k}$ given the message he received and the history of interactions.

All periods have the same weight, 1, except one, the *key* period, period $k^{*}$ (we will assume that $k^{*}=5$), which has weight $\delta _{k^{*}}>1$ (we will assume that $\delta _{k^{*}}\in \{5,10\}$ ).

There are two types of senders. With probability $\alpha$ (in the experiment, $\alpha$ will be either $\frac{1}{10}$ or $\frac{1}{5}$), the sender is *benevolent* and always sends truthful messages,^8^ with probability $1-\alpha$, the sender is *malevolent*. A malevolent sender’s payoff in period *k* is equal to $\delta _{k}(a_{k}-s_{k})^{2}$ and her overall payoff is $\sum \nolimits _{k=1}^{20}\delta _{k}(a_{k}-s_{k})^{2}$. Hence a malevolent sender’s objective is to minimize the receiver’s payoff.

For expositional purposes, we define $d_{k}=|m_{k}-a_{k}|$, the distance between the signal sent by the sender and the decision made by the receiver. Besides, a sender is said to employ a *deceptive* tactic if $m_{k}=s_{k}$ for $k<k^{*}$ and $m_{k^{*}}=1-s_{k^{*}}$. In a deceptive tactic, a sender sends truthful messages before the key period and a false message at the key period.^9^

### Sequential equilibrium analysis

The strategy of the benevolent sender being fixed by the very definition of her type (i.e. sending truthful messages in all periods), a sequential equilibrium of the game is characterized by the strategies of the malevolent sender and the receiver. Since a benevolent sender never sends false messages, by sending one false message, a malevolent sender fully reveals her type. It follows by backward induction, that, in any sequential equilibrium, in all periods following this *revelation*, the malevolent sender sends a truthful message with probability $\frac{1}{2}$ and the receiver chooses action $\frac{1}{2}$. Hence, to characterize a sequential equilibrium, it remains only to determine the strategies of the malevolent sender and of the receiver for histories that do not include a past false message. Such strategies can be summarized for every $k=1, \ldots 20$ by the probability $p_{k}$ that a malevolent sender sends a false message in period *k* conditional on not having sent a false message before, as well as $\hat{d}_{k}$, the value of $d_{k}$ chosen by the receiver in period *k* conditional on not having observed a lie in any prior period. A sequential equilibrium is characterized by the vectors $p=(p_{1},p_{2}, \ldots ,p_{20})$ and $\hat{d}=(\hat{d}_{1},\hat{d}_{2}, \ldots ,\hat{d}_{20})$. We show that there is a unique sequential equilibrium.

#### **Proposition 1**

*For any value of* $(\delta _{k^{*}},\alpha )$, *there is a unique sequential equilibrium characterized by vectors* *p* *and* $\hat{d}$.

#### *Proof*

See the “Appendix”. $\square$

For our experiment, the relevant values of $(\delta _{k^{*}},\alpha )$ are $(5,\frac{1}{5})$, $(10,\frac{1}{5})$ and $(5,\frac{1}{10})$. The corresponding sequential equilibrium can be approximated as follows.

#### **Result 1**

- *For* $$(\delta _{k^{*}},\alpha )=(5,\frac{1}{5})$$, $$p\approx (0.482, 0.458, 0.387, 0.203, 1, \ldots , 1)$$ *and* $$\hat{d}\approx (0.386, 0.309, 0.204, 0.083, 0.354, 0, \ldots , 0)$$.
- *For* $$(\delta _{k^{*}},\alpha )=(10,\frac{1}{5})$$, $$p\approx (0.466, 0.425, 0.329, 0.128, 1, \ldots , 1)$$ *and* $$\hat{d} \approx (0.373, 0.29, 0.181, 0.058, 0.42, 0, \ldots , 0)$$.
- *For* $$(\delta _{k^{*}},\alpha )=(5,\frac{1}{10})$$, $$p\approx (0.497, 0.492, 0.479, 0.435, 1, \ldots , 1)$$ *and* $$\hat{d}\approx (0.447, 0.403, 0.334, 0.237, 0.404, 0, \ldots , 0)$$.

Given the $\hat{d}$ vector, the value of the *p* vector is chosen so that a malevolent sender is indifferent between sending a first false message in any of the first 5 periods. Given the *p* vector, the value of $\hat{d}_{k}$ is the probability of observing a lie in period *k* conditional on not having observed a lie in any prior period. For each parameter value, *p* and $\hat{d}$ as written inside Result 1 can be computed from these conditions.

Roughly, the strategic considerations of this game can de understood as follows. A malevolent sender would like to persuade the receiver that she is benevolent by sending truthful messages during the $k^{*}-1$ initial periods if it allowed her to obtain a high payoff in period $k^{*}$ (a deceptive tactic). If malevolent senders were following a deceptive tactic with high probability, they would not be much trusted in the key period due to the rational expectation assumption in the sequential equilibrium. Besides, their truthful messages during the $k^{*}-1$ first periods of the game would be much trusted so that they would derive low payoffs in these periods. Therefore, this would make the deceptive tactic suboptimal.

This in turn leads the equilibrium frequency of deceptive tactic to be relatively low in equilibrium. Specifically, the *p* vectors introduced in result 1 imply that a malevolent sender always sends her first false message in one of the 5 first periods of the game and chooses a deceptive tactic with probability $\prod \nolimits _{i=1}^{4}(1-p_{i})p_{5}$, which is close to 0.137, 0.18 and 0.075 for $(\delta _{k^{*}},\alpha )$ set at $(5,\frac{1}{5})$, $(10,\frac{1}{5})$ and $(5,\frac{1}{10 })$, respectively. Hence, the frequency of deceptive tactic increases with $\delta _{k^{*}}$ and $\alpha$.

A higher $\delta _{k^{*}}$ makes the deceptive tactic more attractive, since the key period becomes even more important and it is more rewarding to sacrifice payoffs in the $k^{*}-1$ first periods so as to obtain a higher payoff in period $k^{*}$.

When $\alpha$ is higher, it is more likely that the sender is benevolent and therefore, with the same strategy, a malevolent sender can obtain a lower $d_{k^{*}}$. This increases the payoff of the deceptive tactic and its frequency (up to the point where the indifference between choosing a deceptive tactic or sending a first false message is restored).

One may also note that the last $$20-k^{*}$$ periods of the game do not affect the equilibrium behaviors in the first $$k^{*}$$ periods of the game. A malevolent sender always sends her first false message in one of the first $$k^{*}$$ periods and sends a false message with probability $$\frac{1}{2}$$ during the $$20-k^{*}$$ last periods of the game. If we were to consider a variant of the game with only the first $$ k^{*}$$ periods of the game, the sequential equilibrium would be exactly the same as far as these periods are concerned.

### A setup with cognitive limitations

To analyze the data, we consider the analogy-based sequential equilibrium (ABSE) as defined in Ettinger and Jehiel (2010). We consider the following cognitive environment. Both malevolent senders and receivers may be of two different cognitive types. With probability $\beta \in [0,1]$, they are standard rational players and, with probability $1-\beta$, they are (coarse) players not distinguishing their opponent’s behavior as a function of history. Types are private information and independently distributed across players, while $\beta$ is assumed to be known by rational players.

On the sender side, we have in mind coarse players who would fail to appreciate how the future is affected by their current behavior and as a result would randomize 50:50 between telling the truth and lying independently of history. This can be modelled within the ABSE framework by requiring that coarse senders put all the decision nodes of the receivers into one analogy class. For coarse receivers, we have in mind, in agreement with the conditions of the experiment, that they know the aggregate lie rate of the human senders over the 20 periods, but not how their behaviors depend on the history of play.^10^ According to ABSE, coarse receivers are assumed to reason as if human senders were behaving in a stationary way.

In equilibrium, rational players play a best-response to other players’ strategies and coarse players play a best-response to their perceptions of other players’ strategies, using Bayes’ rule to revise their beliefs about the type of player they are matched with.

In order to show how ABSE works, we describe an equilibrium with $\beta = \frac{1}{4}$, assuming that $\alpha =\frac{1}{5}$ so as to match the conditions of the baseline treatment. We choose this specific value of $\beta$ because we will see later that the corresponding ABSE provides a good approximation of the observed experimental data.

#### **Proposition 2**

*There exists an analogy-based sequential equilibrium of the game just defined with* $\beta =\frac{1}{4}$ *and* $\delta _{k^{*}}=5$, *satisfying the following properties:*

- *A coarse malevolent sender uniformly randomizes between sending false and truthful messages during the 20 periods of the game. She sends, on average, 10 false and 10 truthful messages during the 20 periods of the game*.
- *A rational malevolent sender always sends truthful messages during the first 4 periods, sends a false message in period* 5 *and randomizes between truthful and false messages during the last 15 periods of the game. She sends, on average, 10 false and 10 truthful messages during the 20 periods of the game*.
- *In any period* *k* *such that he has never observed a false message in any prior period, a coarse receiver chooses* $d_{k}=\frac{(1-\alpha )(\frac{1 }{2})^{k}}{\alpha +(1-\alpha )(\frac{1}{2})^{k-1}}$. *In any other period, he chooses* $d=\frac{1}{2}$.
- *During the first 4 periods of the game and after having observed at least one false message, a rational receiver mimics the behavior of coarse receivers. Conditional on not having observed a false message during the first 4 periods, a rational receiver chooses* $d_{5}=\frac{(1-\alpha )(\beta +(1-\beta )(\frac{1}{2})^{5})}{(1-\alpha )(\beta +(1-\beta )(\frac{1}{2} )^{4})+\alpha }$ *and, for* $k>5$, *if he did not observe a false message in any prior period, he chooses* $d_{k}=\frac{(1-\alpha )(1-\beta )(\frac{1}{2} )^{k}}{\alpha +(1-\alpha )(1-\beta )(\frac{1}{2})^{k-1}}$.

#### *Proof*

See the “Appendix”. $\square$

The intuition for Proposition 2 is as follows. As already mentioned, coarse senders find it optimal to send a false message with probability $\frac{1}{2}$ in all periods and after any history because they fail to see any link between the messages they send and receivers’ decisions.

Malevolent senders, independently of their cognitive types, send, on average, 10 false messages and 10 truthful messages.^11^ Therefore, coarse receivers have the perception that there are two types of senders with the following characteristics: With probability $\alpha$, senders are *honest* and always send truthful messages and with probability $1-\alpha$, senders are non-trustworthy -we refer to such senders as *liars*- and send truthful and false messages with probability $\frac{1}{2}$ in each period.

When observing at least one false message, a coarse receiver perceives that he is matched with a liar and chooses $d=\frac{1}{2}$ from then on. In period *k*, conditional on having observed only truthful messages in previous periods, a coarse receiver believes that he is matched with a liar with probability $\frac{(1-\alpha )(\frac{1}{2})^{k-1}}{(1-\alpha )(\frac{1}{ 2})^{k-1}+\alpha }$ since he believes that a liar sends a false message with probability $\frac{1}{2}$ in all periods of the game. Therefore, he chooses $d_{k}=\frac{(1-\alpha )(\frac{1}{2})^{k}}{(1-\alpha )(\frac{1}{2} )^{k-1}+\alpha }$ which coincides with his overall perceived probability that the sender sends a false message in the current period given that only malevolent senders lie and they are perceived to lie with probability $\frac{ \mathbf {1}}{2}$. Conditional on only observing truthful messages, $d_{k}$ is strictly decreasing in *k* including in period 5 where $d_{5}=\frac{1}{10}$ if $\alpha =0.2$.

Coarse receivers perceive that a false message in period 5 after 4 truthful messages is quite unlikely (since past behaviors most likely come from a machine). This belief is exploited by rational senders who follow a deceptive strategy with probability 1.

Whenever $\beta$ is not too high, the extra profit that a rational sender makes with coarse receivers is sufficient to compensate for the loss she makes with rational receivers.

Consider next rational receivers. By mimicking the behavior of coarse receivers up to period 4, a rational receiver maintains rational senders in the ignorance of his type, which the rational receiver takes advantage of in the key period 5 by choosing an optimal $d_{5}$ (equal to $\frac{1}{2}$ when $\alpha =\frac{1}{5}$) much higher than $d_{4}$ (equal to $\frac{5}{26}$ when $\frac{1}{5}$). This is better than choosing a myopic best-response in period 1, 2, 3 or 4 because the chance of being matched with a rational sender, i.e. $(1-\alpha )\beta$, is not too small.

For expositional purposes and in order to be consistent with experimental observations, Proposition 2 is stated for $\beta =\frac{1}{4}$. However, a qualitatively similar ABSE would arise for values of $\delta _{k^{*}}$ larger than 5 and for a broad range of values of $\beta$.^12^

During the first 5 periods of the game, behaviors differ significantly in the sequential equilibrium and in the ABSE and the comparative statics for the 20-period version of the game is much simpler in ABSE than in the sequential equilibrium.

Whether $\delta _{k^{*}}=5$ or 10 doest not affect the equilibrium. Coarse players perceive that their opponent plays in the same way independently of the value $\delta _{k^{*}}$ and of the period, therefore, they also play in the same way. Rational senders follow a deceptive tactic with probability 1 when $\delta _{k^{*}}=5$ and are even more willing to do so when $\delta _{k^{*}}=10$. Rational receivers who manage to obtain the highest possible payoff in period $k^{*}$ also adopt the same strategy whether $\delta _{k^{*}}=5$ or 10.

If we lower $\alpha$, again, this does not affect senders’ behaviors. Receivers’ behaviors are not qualitatively modified but the $d_{k}$ conditional on not having observed false message tend to be slightly higher because the probability of being matched with a benevolent sender is lower.^13^

When looking at the version in which the interaction stops at the end of the key period, we note that within our cognitive setup, what we described as an ABSE truncated to the first five periods is no longer an equilibrium. The overall majority of truthful messages from malevolent senders (aggregating over the coarse and rational senders) slightly modifies the receivers’ strategies without affecting the senders’ strategies. As a result, the premium that rational senders obtain when they choose a deceptive tactic is slightly lower when we stop the game after 5 periods.^14^

## Experimental design and predictions

### Elements

The experiment was conducted in the Laboratoire d’Economie Experimentale de Paris, located in the Maison des Sciences Economiques with the software REGATE from 2007 to 2019.The 23 sessions lasted from 1.4 to 1.7 h and the 417 subjects (18 or 19 per session) were predominantly Paris 1 undergraduate students, 40% of them majoring in economics. During the experiments, subjects interacted with each other only through computer terminals. There was no show-up fee, the gains of subjects corresponded exclusively to what they earned from playing the game adding the payoffs from all periods (and all plays of the game, referred to as rounds). Their point payoffs were converted into Euros using a pre-specified exchange rate. Earnings ranged from 8 Euros to 27.80 Euros with a variance of 9.20 Euros and an average of 15.45 Euros. We arranged standard sessions (10 sessions) with $\delta _{k^{*}}=5$ and $\alpha =\frac{1}{5}$ and considered several variants to be described next.

In the baseline treatment (standard sessions), the game was played 5 times (5 rounds), 10 subjects were assigned to the role of receivers and 8 subjects were assigned to the role of senders with a malevolent sender’s utility function as described above. Two computerized machines played the role of benevolent senders.

At the beginning of each round, senders (8 humans + 2 machines) were randomly matched to receivers (10 humans) (stranger matching). Each sender was assigned a capital of false and truthful messages summing to 20. During the game, this capital evolved depending on the number of false and truthful messages sent earlier. During a round, a sender was constantly informed of her remaining capital of false and truthful messages. Whenever her capital of one of the two types of messages was equal to zero, the computer system forced the sender to send the other type of messages until the end of the current round. At the start of an interaction (round), a sender’s capital of false messages was randomly drawn. It could be equal to 9, 10 or 11 with an equal probability for all these draws (this randomness was added to introduce an element of unpredictability toward the end of the game on the receiver side).^15^

Senders and receivers’ instructions contained a complete description of the game except that receivers were not told senders’ utility functions. The framing of receivers’ instructions was however suggestive that the weights attached to the various periods applied both to the Sender and the Receiver.^16^ Receivers were informed that with probability $\frac{4}{5}$ they would be paired with human senders and, with probability $\frac{1}{5}$, with an automaton that always sends truthful messages. They knew that human senders’ strategies were such that they send, on average, 10 false messages and 10 truthful messages across the 20 periods of the baseline treatment.

As mentioned in Introduction, not letting receivers know senders’ payoffs is non-standard in experimental economics, even if it has precedents (see, in particular, Huck et al. 2011 or Esponda and Vespa 2018). One disadvantage is that it makes the Sequential Equilibrium somehow less plausible to the extent that it makes receivers’ guessing about the behavior of senders even more difficult.^17^ An advantage is that it fits better with a number of real life applications in which other players’ payoffs are rarely given from the start and must be inferred from behaviors.^18^

Variants were also considered.

- 10% sessions (3 sessions i.e. 150 rounds). In this treatment, the chance of meeting a truthful machine was reduced from 20% to 10%. This was implemented by having 9 malevolent senders and only one benevolent automaton sender.
- Weight 10 sessions (3 sessions i.e. 150 rounds). In this treatment, the weight of the key period $k^{*}$ was increased to $\delta _{k^{*}}=10$.
- 5 period sessions (3 sessions i.e. 300 rounds^19^ ). In this treatment, the interaction stopped right at the end of the key period $k^{*}$. There was no constraint on the number of false messages. After the first round, receivers were informed of the past aggregate lie rate of human senders.

These first three variants were designed in order to study some comparative statics. The next variant was designed to test the effects of providing receivers with senders’ payoff, as commonly considered in the experimental literature.

- RISP sessions (4 sessions i.e. 200 rounds). This treatment was the same as our baseline treatment except that Receivers were Informed of human Senders’ Payoff (RISP) function. All receivers and senders were gathered in the same room at the same time, the instructions for senders and receivers were communicated and read aloud.

During all sessions, subjects had at their disposal a written version of the instructions and a pencil as well as a piece of paper. Before the beginning of a session, we presented to the subjects the screens that they would have to face during the game. In all sessions, subjects, during a round, could see on the lower part of the screen the history of false and truthful messages of the current round. Instructions appear in the online appendix.

In order to facilitate the computations, the payoffs of the participants of the game were multiplied by one hundred as compared with the game introduced in the previous section.

### Behavioral predictions

The equilibrium analysis that we introduced for the Sequential Equilibrium and the ABSE allows to make behavioral predictions regarding these two solution concepts.

#### Sequential equilibrium

- $P_1$—For $(\delta _{k^{*}},\alpha )=(5,\frac{1}{5})$, the frequency of deceptive tactic should be close to 0.14.
- $P_2$—For $(\delta _{k^{*}},\alpha )=(5,\frac{1}{10})$, the frequency of deceptive tactic should be close to 0.075 (see 10% sessions).
- $P_3$—The frequency of deceptive tactic should increase with the weight of the key period (see weight 10 sessions).
- $P_{4}$—The gain of senders should be the same whether or not they follow a deceptive tactic.
- $P_5$—Conditional on not having observed any false message, receivers should choose $d_{k+1}<d_k$ except for $d_5>d_4$.
- $P_6$—Removing the 15 final periods of the game should not affect behaviors in the 5 first periods of the game (see 5 periods sessions).

#### Analogy-based sequential equilibrium with $\beta =\frac{1}{4}$

- $P_1^{\prime }$—For $(\delta _{k^{*}},\alpha \in \{(5,\frac{1}{5} ), (5,\frac{1}{10}), (10,\frac{1}{5})\}$, the frequency of deceptive tactic should be the same, close to 0.27 (see weight 10 and 10% sessions).
- $P_{2}^{\prime }$—Malevolent senders choosing a deceptive tactic should obtain, on average, a higher payoff than malevolent senders sending their first false message in one of the first 4 periods.
- $$P_{3}^{\prime }$$—Conditional on not having observed any false message, receivers should choose $$d_{k+1}<d_{k}$$ for $$k<4$$. A fraction $$ \frac{1}{4}$$ of receivers (the rational ones) chooses $$d_{5}>d_{4}$$ with $$ d_{5}$$ close to 0.5, and a fraction $$\frac{3}{4}$$ (the coarse receivers) choose $$d_{5}<d_{4}$$.
- $P_{4}^{\prime }$—Removing the 15 final periods of the game should reduce the payoff of malevolent senders following a deceptive tactic without affecting the frequency of deceptive tactic (see 5 periods sessions).

## Results

### First observations in standard sessions

We first describe some salient observations (out of the 500 rounds).

#### The receiver side

We focus on the variable $d_{k}$ rather than $a_{k}$ since what really matters is the distance between the message sent and the action of the receiver.

The average value of *d* over all periods is equal to 0.39 taking into account receivers matched both with benevolent and malevolent senders. If we only consider receivers matched with malevolent senders, this statistic is equal to 0.45, slightly less than 0.5. As it turns out, the distribution of *d*s is heavily affected by a very simple statistic: did the receiver already observe a false message during the game or not?

Conditional on no false message being observed, the average $$ d_{k}$$ slowly decreases from period 1 to 5 (from 0.33 to slightly more than 0.285), decreases faster from period 6 to 9 and reaches 0.11, then again slowly decreases with some oscillations around 0.075. Even after 15 or 18 truthful messages, the average $$d_{k}$$ never falls below 0.06.

**Fig. 1:**
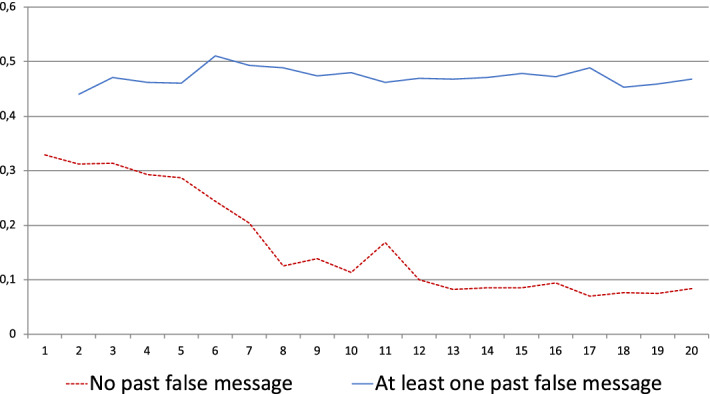
Average *d* in standard sessions

If at least one false message has been observed during the game, the average $d_{k}$ is equal to 0.475. It does not vary much with the period *k*. These observations are gathered in Fig. 1.

The contrast between the distribution of *ds* according to whether or not a lie was previously observed is very much in line with some basic Bayesian understanding of the problem to the extent that a single lie perfectly reveals that the sender cannot be a machine consistently sending truthful messages. The distribution of *d* after a lie is also broadly consistent with the theories presented above ($d=0.5$) even if the data are noisier than according to the theories.

**Fig. 2:**
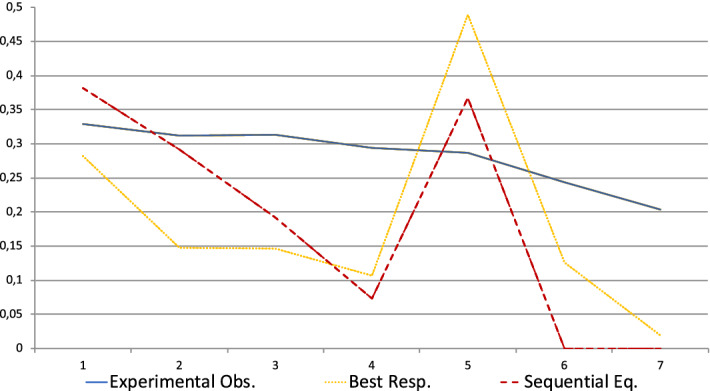
Average *d* (no false message observed) in standard sessions

The downward sloping pattern of $d_{k}$ including at the key period $k^{*}$ when no lie is observed is not consistent with the sequential equilibrium prediction. Conditional on no false message being observed during the game, receivers tend to choose values of $d_{k}$ higher than the ones that would maximize their payoff given the actual behavior of senders. $d_{k}$ decreases too slowly across periods. However, there is one major exception: period 5, the key period. Conditional on having observed only truthful messages during the 4 first periods, receivers should choose a $d_{5}$ much above $d_{4}$ (again considering both actual senders’ behaviors and the sequential equilibrium). However, this is not the case. The average $d_{5}$ is very close to the average $d_{4}$. These observations are represented in Fig. 2 together with the corresponding evolution of $d_{k}$ according to the Sequential Equilibrium (SE).

#### The sender side

The more salient observation on the sender side concerns the deceptive tactic which is chosen with a 0.28 frequency by human senders as compared with the 0.14 frequency of SE.^20^ We note that choosing such a deceptive tactic is much more profitable as compared with the other used strategies (aggregating over the latter) during the 5 first periods of the game. A sender who sends her first false message during one of the 4 first periods of the game obtains on average 292 during the 5 first periods. When she follows a deceptive tactic, she obtains, on average, 361.^21^ This difference is highly significant if we gather the data from all rounds ($p<10^{-4}$, $n=212$, signed-rank test). Now, if we consider each round separately, the difference is significant in rounds 1 and 3 ($p<0.003$, $n_1=32$ and $n_3=47$, signed-rank test).

In order to take into account the possible issue of non-independence of observations, we also ran some tests taking sessions as the unit of observation. For each of the 10 sessions, we consider a pair of observations with the average payoff obtained by senders following the deceptive tactic and the average payoff obtained by senders choosing a different tactic. With a matched-pairs Wilcoxon signed-rank test,^22^ we tested whether we can reject the hypothesis that these two sets of tactics generate the same distribution of payoffs based on these 10 pairs. We obtain a probability $$ p<0.01$$ that these payoffs could be generated by the same distribution, thereby confirming the statistical significance of the superior payoffs obtained with the deceptive tactic.

For the frequency of deceptive tactic, we also ran a bilateral signed-rank test. However, for each session, we only have one observation, the actual frequency of deceptive tactic. We compare this observation to the result of a binomial draw with $n=40$ and $p=0.14$ (what we would obtain if senders were following the SE). We repeat these draws 10 times in order to obtain 10 values that we can compare to the observations obtained in the 10 sessions. We iterated the process 12 times in order to obtain 12 tests of the difference between observations and the predictions of the SE. In all the draws, the difference was significant ($p<0.05$ in all cases, $p<0.02$ in 11 cases and $p<0.01$ in 8 cases).

We also ran a signed-rank test comparing the observed data and the predictions of the ABSE with $\beta =1/4$. We could not reject the hypothesis of an identical distribution, $p\approx 0.62$.

### First interpretations

#### The sender side

Neither the high frequency of observed deceptive tactic nor the extra profitability of this tactic is consistent with the predictions of the sequential equilibrium. We note in our experimental data that a receiver has a higher chance of facing a human sender who employs a deceptive tactic than of facing a machine, which, even without getting into the details of the sequential equilibrium, is at odds with the predictions of the rational model. Moreover, a significant difference in the average revenue obtained with different tactics chosen with positive probability by senders is hard to reconcile with an interpretation in terms of rational agents playing a sequential equilibrium with mixed strategies. In a sequential equilibrium, the tactics chosen with strictly positive probability are supposed to provide the same expected payoff.

As already suggested, we intend to rationalize our data based on the ABSE concept. Of course, allowing ourself to vary the share $\beta$ of rational players in ABSE gives one more degree of freedom in ABSE as compared with SE, and it is thus not surprising that ABSE with well chosen $\beta$ can explain data better than SE. But, our main challenge will be to suggest that such an ABSE with the same share $\beta$ of rational players both on the sender and the receiver sides explains the qualitative features of the complex strategies of the senders and the receivers in the baseline treatment and in a number of variants. Coming back to the observed data, given the proportion 0.28 of observed deceptive tactic, the required proportion $\beta$ of rational subjects should satisfy $\beta +\frac{ 1-\beta }{32}=0.28$.^23^ That is, $\beta \approx 0.25$, hence the choice of $\beta$ in Proposition 2.

**Fig. 3:**
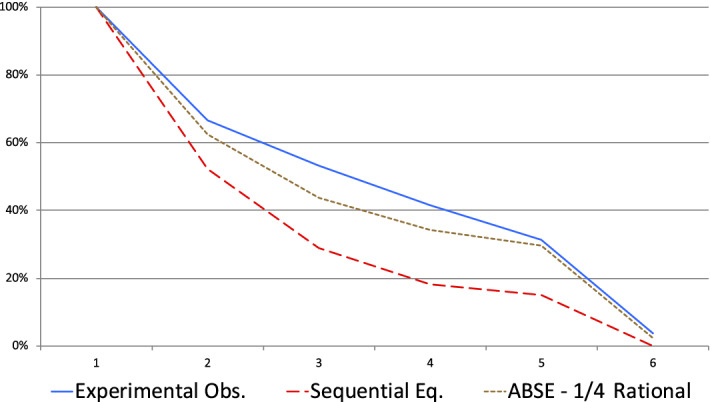
Percentage of malevolent senders having sent only truthful messages at the beginning of the period—Standard sessions

For periods 1 to 4 we compare the proportions of lies conditional on not having observed any lie previously in the data with the ABSE ($\beta =0.25$) and SE theoretical benchmarks. These are depicted in Fig. 3 where we observe a good match between the observed data and ABSE with $\beta =0.25$.

Apart from the good match of lie rate between observations and ABSE, it should also be mentioned that the extra profitability of the deceptive tactic observed in the data agrees with the ABSE prediction.^24^

#### The receiver side

On the receiver side, we wish to explore whether the observed data fit the ABSE with $\beta =1/4$ described in Proposition 2.

For the categorization of receivers into cognitive types, we employ a methodology that retains a salient feature that differentiates the strategies of rational and coarse receivers.

Specifically, coarse receivers as considered above believe that human senders are equally likely to send a false message in each period (independently of the round history). As a result, coarse receivers get more and more convinced that they are facing a machine as they observe no lie in the past with nothing special happening at the key period. Thus, the pattern of $d_{k}$ for coarse receivers is such that $d_{k}$ declines up to and including at the key period, as long as no lie is observed resulting in a $\backslash$-shape for $d_{k}$.

As far as rational receivers are concerned, they are ones who anticipate that the lie rate may be quite high at the key period if no lie has been observed so far (because human senders who have not yet lied are expected to lie at the key period). For rational receivers, as long as no lie has been observed, their $$d_{k}$$ declines up to period $$k^{*}-1$$ and goes up at $$ k^{*}$$ resulting in a V-shape for $$d_{k}$$.

Accordingly, we categorize receivers who have observed no lie from period 1 to 4 into two subpopulations:^25^$\backslash$-receivers and V-receivers. A receiver is a $\backslash$-receiver (identified as a coarse receiver) if, conditional on having only observed truthful messages in the past, he follows more and more the sender’s recommendation up to and including at the key period, or, in symbols, for any $k<5$, $d_{k+1}\le d_{k}$. A receiver is a V-receiver (identified as a rational receiver) if, conditional on having only observed truthful messages in the past, he follows more and more the recommendation before the key period but becomes cautious at the key period, or in symbols, for any $k<4$, $d_{k+1}\le d_{k}$ and $d_{5}>d_{4}$.^26^

**Fig. 4:**
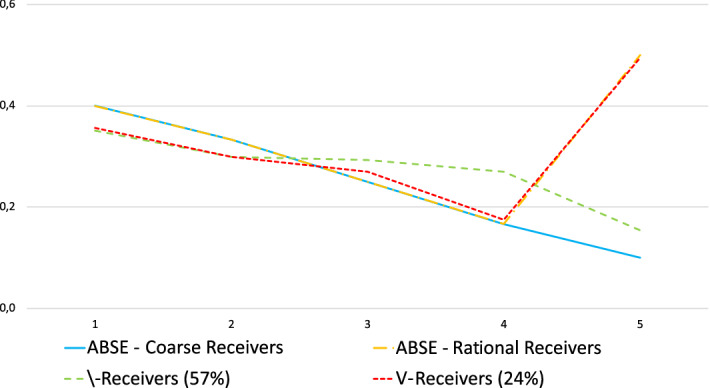
Average d—No past false message—Standard sessions

We observe that most of the receivers who have observed no lie up to period 4 belong to one of these two categories. 57% of the receivers are $\backslash$-receivers and 24% are V-receivers (out of the 212 observations). Retaining a share $\beta =0.25$ of rational subjects as in Proposition 2, Fig. 4 reveals that the average behaviors of these two populations are quite well approximated by identifying $\backslash$-receivers with coarse receivers playing the analogy-based sequential equilibrium and V-receivers with rational receivers playing the ABSE rational receiver’s strategy.^27^ The observed coefficient of the slope slightly differs from the equilibrium predictions but this may be the result of receivers’ difficulties in applying an exact version of Bayes’ law.

Given our suggestion that V-receivers can be thought of as being more sophisticated than $\backslash$-receivers, it is of interest to compare how these two groups performed in terms of their expected gains. The average expected gain over the first five periods is 615 for $\backslash$-receivers and 695 for V-receivers, thereby resulting in a difference of 79 in expected payoff (the prediction of the ABSE is a difference of 80) which is significantly different from 0 in all rounds ($p<0.05$ in round 1 and in all the other rounds $p<0.002$, with *n* varying from 32 to 52 depending on the rounds, paired T-test). We also ran a signed-rank test following the same procedure as for the payoff comparison of senders (following or not a deceptive tactic). The difference of payoffs in these two groups of receivers is significant with $p<0.06$.

As just reported, our analysis of the baseline treatment suggests that the experimental data are well organized by the ABSE shown in Proposition 2 with a $\beta =\frac{1}{4}$ share of rational subjects and $\frac{3}{4}$ share of coarse subjects both on the sender and the receiver sides. In order to improve the fit, one could allow subjects to use noisy best-responses as in Quantal Response Equilibrium models, but the computation of the corresponding ABSE is quite complicated, which has led us not to follow this route. In an attempt to allow for noisy behavior, in the “Appendix”, we develop a statistical method for the categorization of receivers into rational vs coarse types, explicitly allowing for mistakes and focusing on types who would either if rational take the actual aggregate lie behavior (conditional on no lie being observed so far) as their belief or else they would consider that the lie rate is uniformly 50:50 for human senders exactly as coarse receivers would do in ABSE. The method assigns each individual to one or the other type according to the likelihood for each type to generate their observed behavior. The results obtained with this alternative method are qualitatively very close to those obtained with the $\backslash$-receivers and V-receivers categorization. They are reported in the “Appendix”.

### Variants

We now discuss the experimental findings in the various variants we considered.^28^

#### 10% automata/ weight 10

First note that in the $10\%$ automata case ($\alpha =\frac{1}{10}$), with $\beta$ unchanged, the ABSE is the same as in Proposition 2 (with values of $d_{k}$ adjusted to the changes of $\alpha$). The SE has the same properties as when $\alpha =\frac{1}{5}$ with a much lower frequency of deceptive tactic ($\approx 0.075$), since a smaller $\alpha$ makes it less profitable for a malevolent sender to be confounded with a machine.

Experimental observations with $10\%$ automata are almost identical to the ones we obtained in the baseline treatment: A ratio 0.25 of deceptive tactics and of V-receivers. This comparative statics is consistent with the ABSE and a share $\beta =\frac{1}{4}$ of rational subjects, much less with the sequential equilibrium.

If we increase the weight $\delta _{k^{*}}$ of the key period from 5 to 10, this increases the frequency of deceptive tactics in the sequential equilibrium and, ceteris paribus, does not affect the ABSE with $\beta =\frac{1}{4}$ shown in Proposition 2.

In the data of the weight 10 sessions, the frequency of deceptive behavior is slightly lower than in the baseline treatment (0.19) and the ratio of V-receivers slightly higher ($30\%$). The relative stability and the direction of the slight modification of these frequencies is more in line with our interpretation in terms of ABSE with a share $\beta =\frac{1}{4}$ of rational subjects than an interpretation in terms of subjects playing SE. Maybe the slight difference with the baseline case can be interpreted along the following lines. As the weight of the key period increases, it becomes more salient, thereby leading receivers to pay more attention to it, which may result in a higher ratio of V-receivers. Anticipating this effect, rational senders are less eager to follow a deceptive strategy which is less likely to be successful.

#### RISP sessions

In these sessions, both the senders and the receivers were informed of the payoff functions of all the participants, as more commonly considered in experimental economics. We find that the frequency of deceptive tactic is close to 0.19 with a slightly increasing trend (the frequency is equal to 0.21 in the last two rounds). On average, senders obtain a higher payoff when they follow a deceptive tactic as compared with any other tactic. If we aggregate all rounds, they obtain 317 as compared to 290. The difference is significant ($p\approx 0.01$, signed-ranked test, $n=103$). The fraction of $\backslash$-receivers (resp: V-receivers) is $40\%$ (resp: $25\%$).

**Fig. 5:**
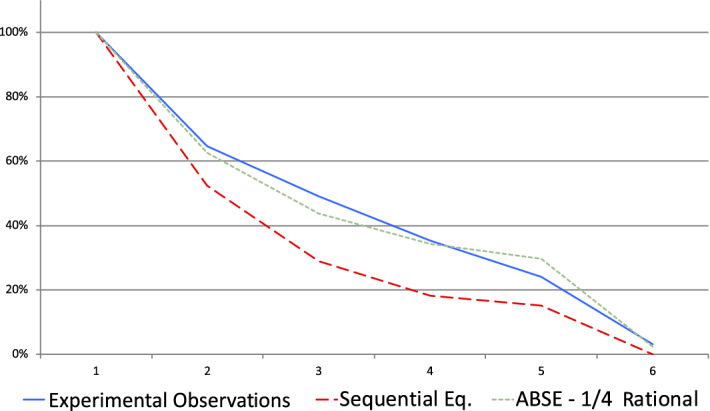
Percentage of malevolent senders having sent only truthful messages at the beginning of the period—RISP sessions

Observations do not coincide with the predictions of the SE. On the receiver side, $P_{5}$ predicts that all receivers should be V-receivers who represent in the data only $25\%$ of all receivers (while $40\%$ are $\backslash$-receivers). On the sender side, the frequency of deceptive tactic is higher than according to the SE predictions with a significant difference ($p<0.02$, $n=160$, T-test). Figure 5 illustrates that if we consider more generally the average behaviors of senders during the first 5 periods of the game, ABSE (with $\beta =\frac{1}{4}$) provides a much better explanation of the data than SE. Moreover, senders obtain a much higher payoff when they follow a deceptive tactic than when they do not: 675 rather than 403 ($p<10^{-2}$, signed-rank test, $n=103$).

With this variant, it is also particularly instructive to disentangle the observations in the different rounds. In rounds 1 and 2, the deceptive tactic gives average payoffs of 375 and 386 respectively as compared to 298 and 257 for non-deceptive tactics. The difference is significant ($p<0.034$ and $p<0.02$, signed-rank test, $n_1=17$ and $n_2=21$). In rounds 3, 4 and 5, the deceptive tactic gives average payoffs close to what is obtained with non-deceptive tactics. The difference is no longer significant ($p\in (0.44,0.94)$, signed-rank test, $n=22$ or $n=21$).

This shows that there is a clear-cut learning process at work in RISP sessions (we will elaborate on the learning dimension on the receiver side later on). From a different perspective, observe that the similarity in the observed behaviors in the first two rounds of RISP and in the five rounds of the baseline sessions is suggestive that coarse receivers only initially use heuristics in the vein of ABSE in RISP. That is, when other player’s preferences are known, the type of reasoning in ABSE prevails more (less) with less (more) experienced subjects.

#### 5 period sessions

The predictions of the sequential equilibrium in this variant are the same as in the standard treatments. However, in this variant, we observe very different behaviors. On the sender side, the frequency of deceptive tactic is equal to 0.05 (out of 240 observations), much lower than in any other variant and much lower than predicted by the sequential equilibrium or the ABSE with $\beta =\frac{1}{4}$

On the receiver side, we observe higher *ds*. Except between periods 1 and 2, the average $d_{k}$ conditional on having observed only truthful messages is decreasing in *k* but the values are higher than in all the other variants, between 0.44 and 0.38 (in period 5). The shares of $\backslash$-receivers and V-receivers are 58% and 18%, respectively.

In general, behaviors are explained neither by SE nor by ABSE with $\beta = \frac{1}{4}$ (accounting in ABSE for the fact that the key period is final). On the sender side, behaviors seem to be better explained assuming that in every period, human senders would randomize 50:50 between telling the truth and lying.^29^ On the receiver side, even rational subjects should behave like $\backslash$-receivers if they were rightly perceiving the high share of coarse subjects on the sender side. Receivers behaviors are explained neither by SE nor by ABSE.

Based on the above, it is fair to acknowledge that the set-up with cognitive limitations that we suggest does not provide a good explanation for the data in the 5 period sessions. The complexity of the game also differs, which may be the reason why subjects reason differently.

#### Learning process on the receiver side?

In this part, we ask ourselves whether there is some learning trend on the receiver side. Specifically, we analyze whether there is a change across rounds in terms of the share of $\backslash$-receivers and V-receivers.

Consider first the baseline treatment. We observe that the percentage of $\backslash$-receivers is identical in rounds 1 and 2 and in round 5: 56%. This suggests that there is no clear learning trend at this aggregate level. We next turn to whether receivers’ behaviors depend on their specific history. More precisely, we consider separately two different subsets of histories. H1: The receiver has never been matched with a deceiving sender (i.e. a malevolent sender who used a deceptive tactic against him). H2: The receiver has already been matched at least once with a deceiving sender.

Now, let us consider rounds 4 and 5. In H1 (resp: H2), the frequency of $\backslash$-receivers is 74% (resp: 48%) and the frequency of V-receivers is 3% (resp: 30%). The difference of frequencies observed in the two sets of histories is highly significant ($p<0.064$, $n=21$ and $p<0.01$, $n=12$ respectively, signed-rank test). Receivers are more likely to be V-receivers (rational) if they have been previously matched with a deceiving sender, thereby suggesting that with sufficiently many repetitions, a larger share $\beta$ of rational subjects would be required to explain observed behaviors with ABSE.

It is worth noting that the learning process seems to be even faster in RISP sessions. The frequency of $\backslash$-receivers (resp: V-receivers) among receivers who have been matched at least once with a deceiving sender is 17% (resp: 57%) while the frequency of $\backslash$-receivers (resp: V-receivers) among receivers who have never been matched with a deceiving sender is 46% (resp: 16%). If we only consider rounds 4 and 5, these statistics are respectively equal to 13%, 60%, 42% and 17% and, although the sample is pretty small, the difference is significant for the frequency of $\backslash$-receivers and V-receivers ($p\approx 0.06$ and $p<0.01$ respectively, $n_1=15$ and $n_2=29$, T-test). These results about receivers in RISP are consistent with the observations we made on the evolution of the profitability of the deceptive tactic.

Overall, our analysis reveals some learning effect, after being exposed to a deceptive tactic. This effect is more pronounced in RISP sessions, presumably because the knowledge of the other party’s preferences allows to better make sense of the observation of a deceptive tactic in this case.

Understanding more completely the learning model used by receivers when exposed repeatedly to our baseline 20-period game goes beyond the scope of this paper. But, the kind of deception as modeled in Ettinger and Jehiel (2010) is primarily designed for describing inexperienced receivers who would not have been exposed to deception so far and would base their inference process on a simplified representation of strategies as resulting from the coarse statistical knowledge they have access to. Given this, it is not so surprising that the ABSE framework would have more bite when receivers are less experienced.

#### Summary

None of the 6 predictions introduced in Sect. 3.2 concerning the SE ($P_{1}$ to $P_{6}$) is observed in the data. The frequency of deceptive tactic is much higher than 0.14 (resp: 0.075) in the baseline treatment (resp: 10% automata sessions). The frequency of deceptive tactic slightly decreases with the weight of the key period. Senders obtain a higher payoff on average when they follow a deceptive tactic (except in the final rounds of the RISP sessions as well as the 5 period sessions for which the difference is not significant. The ratio of V-receivers is never higher than $30\%$ in any treatment. Removing the 15 final periods of the game does affect behaviors in the first 5 periods of the game.

The predictions of ABSE are much more in line with the experimental data.

$P_1^{\prime }$—We do observe a frequency of deceptive tactic close to 0.28 with the baseline treatment and 10% automata sessions. The frequency is closer to 0.2 both for RISP and weight 10 sessions and the trend is slightly increasing.

$P_2^{\prime }$—Malevolent senders choosing a deceptive tactic do obtain a higher payoff except in the last round of the RISP sessions (and the difference is not much significant in the 5 periods sessions).

$P_{3}^{\prime }$—With some variations, the frequency of V-receivers in the different treatments is generally quite close to 0.25 and the frequency of $\backslash$-receivers lies between 0.4 and 0.6, in line with ABSE predictions.

$P_{4}^{\prime }$—Removing the 15 final periods of the game does affect behaviors to a much greater extent than predicted. ABSE does not organize the data well in the 5 period sessions.

Except for the 5 period sessions, results are well organized by ABSE assuming that the share of rational subjects is $\beta =\frac{1}{4}$ both on the sender and the receiver sides. By contrast, in the 5 period sessions, neither ABSE nor SE organize the data well. The cognitive assumptions of ABSE may be less suited to this *simpler* version of the game.

## Conclusion

We have reported results from experiments on multi-period sender-receiver games in which one period has a significantly higher weight. We have observed that players’ behaviors are not well captured by the sequential equilibrium of the game at least when players are not too experienced. More precisely, senders tend to follow deceptive tactics (i.e. sending truthful messages until the key period and a false message at the key period) with a much higher frequency than what the sequential equilibrium of the game would predict. Moreover, deceptive tactics provide a higher payoff than other chosen tactics.

We suggest that the high frequency of deceptive tactics as well as their success can be explained by a different equilibrium concept, the analogy-based sequential equilibrium (ABSE). Observations favor the idea that both senders and receivers are heterogenous in their cognitive abilities, some share (roughly $\frac{3}{4}$) employing a coarse reasoning with a smaller share (a quarter) employing a more sophisticated mode of reasoning. Our observations are robust to the introduction of several modifications of the game (notably a change in the share of non-human senders or a change in the weight of the key period) but not in the variant in which the game ends at the key period (in which senders seem to be excessively afraid of using the deceptive tactic and instead seem to be playing randomly).

Our experimental findings suggest that we should see more deceptive tactic when the interaction is not stopped right after the time at which stakes are higher which may fit better in contexts in which communication stages occur in pre-arranged ways. Moreover, putting aside the findings in the 5 period sessions (for which theories beyond those considered here are needed), our study suggests that solution concepts allowing for coarse reasoning may fruitfully be used to shed light on deception, where a closer look at our data reveals that coarse reasoning is more widespread when subjects are less experienced given that an exposure to a deceptive tactic was shown to shift behavior toward that of rational types. Even if coarse reasoning becomes less prominent with experience, deception of the type highlighted here is of practical importance given that experienced agents keep being replaced by less experienced agents in the real world (and yet the novice agents are still exposed to past aggregate statistics, thereby making the equilibrium approach of ABSE compelling for this case).

### Electronic supplementary material

Below is the link to the electronic supplementary material.

Supplementary material 1 (pdf 128 KB)

## Appendix

### Alternative approaches

We review here alternative approaches to see how well they can explain our experimental findings.

Alternative popular approaches to study experimental data include the Quantal Response Equilibrium (see McKelvey and Palfrey 1995) and the level-k approach (following Stahl and Wilson 1994, 1995 and Nagel 1995).^30^

While it is too complex to characterize the QRE in the context of our game with a continuum of actions (on the receiver side), we make the following observations. First, we conjecture that the share of deceptive tactics would not be higher in the QRE than in the sequential equilibrium (think of the extreme version of QRE in which strategies would not be responsive to payoffs in which case the deceptive tactic would appear with probability 3%). Second, considering the data, we observed that the deceptive tactic was more rewarding when the weight of the key period was 10 instead of 5. Yet, the share of deceptive tactics was not bigger in this treatment (it was in fact slightly smaller). These observations are not suggestive that QRE provides a good account of observed data.

Regarding level-k, we observe that the similarity of observations in the first two rounds of RISP and the main treatments is not supportive of the level-k view according to which the opponent’s payoff structure is used to simulate what you would do in the shoes of the opponent. Concerning the formalism of level-k in extensive form games, we note that there is no consensus on how to define it essentially because it requires specifying how level-k beliefs should be revised once an inconsistent behavior is observed.^31^ In an attempt to bypass these difficulties, we consider below one possible level-k approach of our game viewed in normal form assuming that level-0 (human) senders randomize between telling the truth and lying with probability 50:50 in every period, and that level 0 receivers always trust what they are told, thereby choosing $d_{k}=0$ in all periods *k*.^32^

With this specification, level-1 senders would use a deceptive tactic, level-1 receivers would behave as our coarse receivers, level-2 senders would again use a deceptive tactic, level-2 receivers would choose $d_{k}=0$ for $k=1,\ldots 4$ and $d_{5}=0.8$ (anticipating a deceptive tactic on the sender side), level-3 senders would tell the truth up to and including the key period (anticipating that the deceptive tactic is what receivers expect), level-3 receivers would behave like level-2 receivers, and the behaviors of senders and receivers would cycle for higher levels.

Such an approach does provide some account of our observations (the deceptive tactic appears as a possible focal behavior). But, it makes no prediction as to whether the deceptive tactic should be profitable given the realized actions (in contrast to ABSE in which the deceptive tactic is chosen by rational senders precisely because it is profitable in this sense). Moreover, the other (focal) behaviors emerging from the approach do not show up in our data (for example, we see almost no human sender telling the truth up to and including the key period nor do we see receivers choosing $d_{5}\approx 0.8$ after 4 truthful messages, which would be the best-response to the belief that senders follow the deceptive tactic). Moreover, like the sequential equilibrium, the level-k approach predicts that there should be no difference between the 5 period sessions and our main treatments, which is not so in our data.^33^

In the rest of this section, we briefly consider additional approaches one can think of (these are not so natural given the way the instructions were provided to subjects, but they are natural to consider from a conceptual viewpoint). First, senders and receivers may be allowed to entertain subjective beliefs regarding the share of benevolent senders. We note that varying these subjective beliefs would not allow to explain the large presence of $\backslash$-receivers (SE with different shares of benevolent senders would predict V-patterns for receivers).^34^ Second, senders and receivers may be allowed to be inattentive to the fact that period 5 has a higher weight. We note that such a variation would have a hard time explaining the observed share of deceptive tactic which is already much beyond the one predicted by SE with the correct weight on the key period (and a fortiori even further away from the one associated with SE and a smaller weight on the key period).b

### Proof of Proposition 1

By definition, benevolent senders send truthful messages. Moreover, it is clear (by backward induction) that once a lie has been observed (so that it is common knowledge that the sender is malevolent), the sender and the receiver play as in the unique Nash equilibrium of the stage game (i.e., the sender randomizes 50:50 between telling the truth and lying, and the receiver chooses $d=0.5$).

Therefore a sequential equilibrium is characterized by *p* and $\hat{d}$. Besides, $\hat{d}$ being a best response to *p* it is uniquely defined as follows. When $k\ne 1$, the conditional probability that the sender is malevolent is: $\frac{(1-\alpha )\prod _{i=1}^{k-1} (1-p_{i})}{(1-\alpha )\prod _{i=1}^{k-1} (1-p_{i})+\alpha }$ and since, by definition, the probability that a malevolent sender will choose a false message is $p_k$, the receiver’s best response is $\hat{d}_{k}=\frac{ (1-\alpha )\prod _{i=1}^{k-1} (1-p_{i})p_{k}}{(1-\alpha )\prod _{i=1}^{k-1} (1-p_{i})+\alpha }$. When $k=1$, the best response is $(1-\alpha )p_k=\hat{d} _1$. $\hat{d}$ being defined once *p* is fixed, a sequential equilibrium is uniquely characterized by the vector *p*.

Now, it remains to prove the uniqueness of the equilibrium vector $(p_{1}, \ldots ,p_{20}$).

Suppose that $(p_{1}, \ldots ,p_{20})$ and $(q_{1}, \ldots ,q_{20})$ are two different equilibrium vectors.^35^ We define $\widetilde{k}$ such that $p_{ \widetilde{k}}\ne q_{\widetilde{k}}$ and $\forall$ *k* such that $k< \widetilde{k}$, $p_{k}=q_{k}$. We also assume, without loss of generality that $q_{\widetilde{k}}>p_{\widetilde{k}}$.

We introduce $$k_{q}^{r}$$ (resp: $$k_{p}^{r}$$), the revelation period, defined as follows. For any integer $$i<k_{q}^{r}$$ (resp: $$i<k_{p}^{r}$$), $$q_{i}<1$$ (resp: $$p_{i}<1$$) and $$q_{k_{q}^{r}}=1$$ (resp: $$p_{k_{p}^{r}}=1$$) or $$ k_{q}^{r}=21$$ (resp: $$k_{p}^{r}=21$$). We also denote $$\hat{d}_{q}$$ (resp: $$ \hat{d}_{p}$$), the equilibrium $$\hat{d}$$ chosen by receivers in an equilibrium with a *q*-vector (resp: *p*-vector).

Let us compare the equilibrium payoff of a malevolent sender sending her first false message in period $\widetilde{k}$ with both types of equilibrium, a *p*-equilibrium and a *q*-equilibrium. In any period $k<\widetilde{k}$, since $p_{k}=q_{k}$, the best response of the receiver is the same and the payoff is the same for a malevolent sender sending a truthful message either in a *p*-equilibrium or in *q* -equilibrium. In any period *k* after a false message in period $\widetilde{k }$, the receiver chooses $d_{k}=1/2$ and the payoff is the same for the malevolent sender who has sent a false message in period $\widetilde{k}$ either in a *p*-equilibrium or in *q*-equilibrium. Now, in period $\widetilde{k}$, in a *p*-equilibrium, the receiver chooses a $\hat{d}_{ \widetilde{k},p}$ equal to $\frac{(1-\alpha )\prod _{i=1}^{k-1}(1-p_{i})p_{ \widetilde{k}}}{(1-\alpha )\prod _{i=1}^{k-1}(1-p_{i})+\alpha }$ and in a *q* -equilibrium, the receiver chooses a $\hat{d}_{\widetilde{k},q}$ equal to $\frac{(1-\alpha )\prod _{i=1}^{k-1}(1-q_{i})q_{\widetilde{k}}}{ (1-\alpha )\prod _{i=1}^{k-1}(1-q_{i})+\alpha }$ and since $\frac{ (1-\alpha )\prod _{i=1}^{k-1}(1-p_{i})}{(1-\alpha )\prod _{i=1}^{k-1}(1-p_{i})+ \alpha }=\frac{(1-\alpha )\prod _{i=1}^{k-1}(1-q_{i})}{(1-\alpha ) \prod _{i=1}^{k-1}(1-q_{i})+\alpha }$, $\hat{d}_{\widetilde{k},p}<\hat{d}_{ \widetilde{k},q}$ so that the payoff obtained by a malevolent sender sending her first false message in period $\widetilde{k}$ is strictly higher in a *p*-equilibrium than in a *q*-equilibrium. Then, because of the properties of the mixed equilibrium, a malevolent sender always obtains a strictly lower payoff in a *q*-equilibrium than in a *p*-equilibrium.^36^

We intend to show, by induction, that for any $i \in [\widetilde{k},k_q^r]$, $p_i=q_i=0$ or $p_i<q_i$ and $\hat{d}_{i,p}<\hat{d}_{i,q}$.

First, we observe that this property is verified for $i=\widetilde{k}$. Now, suppose that for any $i\in [\widetilde{k},\bar{k}]$ with $\bar{k}$ such that $\widetilde{k} \le \bar{k}<k_q^r$, $p_i=q_i=0$ or $p_i <q_i$ and $\hat{d} _{i,p}<\hat{d}_{i,q}$. Let us first observe that, since for any $i\in [ \widetilde{k},\bar{k}]$, $p_i=q_i=0$ or $p_i <q_i$, $\bar{k}<k_p^r$. Now, suppose that $p_{\bar{k}+1},q_{\bar{k}+1}>0$ and let us consider a malevolent sender sending her first false message in period $\bar{k}+1$. She obtains the same payoff in all the periods whether she plays a *p* -equilibrium or a *q*-equilibrium except in periods from $\widetilde{k}$ to $\bar{k}+1$. In these periods, in a *p*-equilibrium, she obtains $\sum _{j=k}^{ \bar{k}}\delta _j \hat{d}_{j,p}^2+\delta _{\bar{k}+1}(1-\hat{d}_{\bar{k} +1,p})^2$ and in a *q*-equilibrium, she obtains $\sum _{j=k}^{\bar{k} }\delta _j \hat{d}_{j,q}^2+\delta _{\bar{k}+1}(1-\hat{d}_{\bar{k}+1,q})^2$. Besides, for any $j\in [\widetilde{k},\bar{k}]$, $\hat{d}_{j,p}^2 \le \hat{d} _{j,q}^2$, this inequality being strict at least for $j=\widetilde{k}$. Because of the indifference in mixed strategies $\sum _{j=k}^{\bar{k} }\delta _j \hat{d}_{j,p}^2+\delta _{\bar{k}+1}(1-\hat{d}_{\bar{k} +1,p})^2>\sum _{j=k}^{\bar{k}}\delta _j \hat{d}_{j,q}^2+\delta _{\bar{k}+1}(1- \hat{d}_{\bar{k}+1,q})^2$. Therefore, $(1-\hat{d}_{\bar{k}+1,p})^2>(1-\hat{d} _{\bar{k}+1,q})^2$ which also implies $\hat{d}_{\bar{k}+1,p}<\hat{d}_{\bar{k} +1,q}$ and $p_{\bar{k}+1}<q_{\bar{k}+1}$.

Now, we need to show that $p_{\bar{k}+1}>0$ and $q_{\bar{k}+1}=0$ is impossible. If this were the case, the payoff of a malevolent sender in the periods between $\widetilde{k}$ and $\bar{k}+1$, in a *q*-equilibrium, if she deviates and sends her first false message in period $\bar{k}+1$ would be $\sum _{j=k}^{\bar{k}}\delta _j \hat{d}_{j,q}^2+\delta _{\bar{k}+1}$. Because of the arguments we have just mentioned $\sum _{j=k}^{\bar{k} }\delta _j \hat{d}_{j,q}^2+\delta _{\bar{k}+1}>\sum _{j=k}^{\bar{k}}\delta _j \hat{d}_{j,p}^2+\delta _{\bar{k}+1}(1-\hat{d}_{\bar{k}+1,p})^2$. Therefore, a malevolent sender deviating in a *q*-equilibrium, sending her first false message in period $\bar{k}+1$ obtains more than a malevolent sender in a *p* -equilibrium. This cannot be possible since we showed that a malevolent sender always obtains a strictly lower payoff in a *q*-equilibrium than in a *p*-equilibrium. Hence $p_{\bar{k}+1}>0$ and $q_{\bar{k}+1}=0$ is impossible. Hence, for any $i \in [\widetilde{k},\bar{k}+1]$, $p_i=q_i=0$ or $p_i<q_i$ and $\hat{d}_{i,p}<\hat{d}_{i,q}$. End of the induction proof.

Now, we use the result we proved with the induction proof.

First, we show that $k_{q}^{r}=21$ or $k_{p}^{r}=21$ is not possible. Suppose that $k_{p}^{r}=21$. In a *p*-equilibrium, a malevolent sender who did not have sent a false message in any prior period must be indifferent between sending a false and truthful message in period 20 since the choice of a truthful or a false message does not affect her payoff in future period.^37^ This means that $\hat{d}_{20,p}=1/2$. By backward induction, we also obtain that $\hat{d}_{19,p}=1/2$, $\hat{d} _{18,p}=1/2$ . But, this is not possible at the equilibrium (because the sequence $p_{k}=\frac{1}{2}\frac{(1-\alpha )\prod _{i=1}^{k-1}(1-p_{i})+\alpha }{(1-\alpha )\prod _{i=1}^{k-1}(1-p_{i})}$ exceeds 1 at some point). The same arguments apply to reject $k_{q}^{r}=21$.

Let us consider $k_q^r<k_p^r<21$ (this is always the case because of the result we proved by induction) and define $\widehat{k}$ as follows: $k_q^r< \widehat{k}$, $p_{\widehat{k}}>0$ and $\forall i$ such that $k_q^r<i< \widehat{k}$, $p_i=0$ ($\widehat{k}$ is the first period posterior to $k_q^r$ in which a malevolent sender sends her first false message with a strictly positive probability in a *p*-equilibrium) . We consider the following deviation in a *q*-equilibrium: send the first false message in period $\widehat{k}$. In all the periods before $\widetilde{k}$, after $\widehat{k}$ and between $k_q^r$ and $\widehat{k}$, the payment is the same with this strategy as what a malevolent sender obtains in a *p*-equilibrium if she sends her first false message in period $\widehat{k}$. In a *q*-equilibrium, the receiver does not expect any false message in period $\widehat{k}$ conditional on not having observed any prior false message so that sending a false message, the malevolent sender obtains $\delta _{\widehat{k}}$ in this period, the highest possible payoff. Besides in any period *i* from $\widetilde{k}$ to $k_q^r$ (including these periods), $\hat{d}_{i,p} < \hat{d} _{i,q}$ or $\hat{d}_{i,p}=\hat{d}_{i,q}=0$ (but this cannot be the case in all the periods) so that a malevolent sender deviating in a *q*-equilibrium sending her first false message in period $\widehat{k}$ obtains strictly more than a malevolent sender in a *p*-equilibrium sending her first false message in period $\widehat{k}$. But we found that a malevolent sender always obtain a strictly lower payoff in a *q*-equilibrium than in a *p* -equilibrium. Hence, the deviation is strictly profitable, the *q* -equilibrium is not valid and we can reject the possibility of multiple equilibria. $\square$

### Proof of Proposition 2

First, we need to describe more completely the strategies that we only partially introduced in proposition 2 for rational senders.

In case she observes a $d_k$ different from $\frac{4(1/2)^k}{1+4(1/2)^{k-1}}$ in period $k=1,$ 2,  3 or 4, she plays as in the sequential equilibrium of a variant of the game beginning in period $k+1$, with a fraction $\frac{ 2^k}{3+2^{k+1}}$ of benevolent senders, a fraction $\frac{2^k}{3+2^{k+1}}$ of rational malevolent senders and a fraction $\frac{3}{3+2^{k+1}}$ of mechanical senders sending truthful messages with probability 1/2 in each period. Let us also mention that conditional on having observed $d_k=\frac{ 4(1/2)^k}{1+4(1/2)^{k-1}}$ in the 5 first periods of the game, a rational sender sends a false message with probability $\frac{9-\frac{15}{2}\beta }{ 15(1-\beta )}$ during the last 15 periods of the game. If she observes a different vector of $d_k$s during the first 5 periods of the game, she sends a false message with probability $\frac{1}{2}$ in the 15 last periods of the game.

Now let us check that these strategies are constitutive of an ABSE.

A coarse malevolent sender puts all the decision nodes of the receivers in a unique analogy class. Therefore, she does not perceive the link between the message she sends and the decision of the receivers she is matched with. Sending a truthful and a false message with probability $\frac{1}{2}$ in all the periods is a best response with this belief.

Considering senders’ strategies, a rational receiver cannot raise his payoff choosing a $d\ne \frac{1}{2}$ conditional on having observed at least one false message. Therefore, we can focus on his behavior conditional on not having received any false message. A rational receiver must decide in that case whether he mimics coarse receivers or he reveals his type choosing a different *d*. If he reveals her type in period *k*, a coarse sender will continue sending a false message with probability $\frac{1}{2}$ in all the periods and a rational sender will play as in the sequential equilibrium of a variant of the game beginning in period $k+1$, with a fraction $\frac{2^k}{ 3+2^{k+1}}$ of benevolent senders, a fraction $\frac{2^k}{3+2^{k+1}}$ of rational malevolent senders and a fraction $\frac{3}{3+2^{k+1}}$ of mechanical senders sending truthful messages with probability $\frac{1}{2}$ in each period. Therefore, the best response for a rational receiver will be also to play as in the sequential equilibrium of a variant of the game beginning in period $k+1$, with $\frac{2^k}{3+2^{k+1}}$ benevolent senders, $\frac{2^k}{3+2^{k+1}}$ rational malevolent senders and $\frac{3}{3+2^{k+1}}$ mechanical senders sending truthful messages with probability $\frac{1}{2}$ in each period. Now, a rational receiver must choose the period *k* in which he reveals his type and $d_k$. Since the value of $d_k$ does not affect the payoff in the following periods as long as $d_k \ne \frac{4(1/2)^k}{ 1+4(1/2)^{k-1}}$, his best choice is a $d_k$ which maximizes his period expected payoff i.e. if $k<5$, $d_k=\frac{3(1/2)^k}{2+3(1/2)^{k-1}}$, $d_5= \frac{1}{2}$ and if $k>5$, $d_k=\frac{3(1/2)^k}{1+3(1/2)^{k-1}}$. Finding the *k* that maximizes the rational receiver expected payoff is only a matter of computations (requiring to compute expected payoff in the sequential equilibria of all the considered variants of the game). The solution is $k=5$.

Rational senders. Again, the key element is the period of the first false message. After this first false message, in all the remaining periods, $d_k= \frac{1}{2}$, therefore any choice is a best response and she obtains $\frac{ \delta ^k}{4}$ in period *k*. Then, considering the strategies of the different types of receivers, it is only a computation issue to find the best choice for a rational sender. As long as she believes that she is matched with a coarse receiver with probability $\frac{3}{4}$, she obtains a higher payoff sending her first false message in period 5 (her expected payoffs conditional on sending a first false message in period 1, 2, 3, 4 or 5 are respectively and approximatively 2.36, 2.3544, 2.3336, 2.2781 and 3.711), following a deceptive tactic. $\square$

### An alternative statistical analysis

We developed a second methodology based on a statistical model in order to characterize receivers’ behaviors. Given the seemingly noisy character of decisions, we allow subjects to play noisy best-responses using a logit specification which is commonly used in econometrics or in experimental work. That is, if in period *k*, the belief that the sender is lying is *b*, the receiver will choose action *d* with a probability proportional to:^38^

$$\begin{aligned} \exp \lambda V_{k}(b,d) \end{aligned}$$

where

$$\begin{aligned} V_{k}(b,d)=\delta _{k}[1-b(1-d)^{2}-(1-b)d^{2}] \end{aligned}$$

denotes the expected period *k* utility of playing *d* given the belief *b* and $\lambda$ denotes a noise parameter to be estimated (the higher $\lambda$ the less noisy the best response).

The two types coarse and rational of receivers correspond to different specifications of *b*. The belief of a coarse receiver in period *k* when no lie was observed is given by:

$$\begin{aligned} b_{k}^{c}=\frac{(1-\alpha )(1/2)^{k}}{\alpha +(1-\alpha )(1/2)^{k-1}} \end{aligned}$$

given that coarse receivers expect human senders to lie with probability $\frac{1}{2}$ in every period (and they know that honest senders always tell the truth).

Regarding non-coarse receivers, we considered several variants for $b^{r}$,^39^ but picking the one that gave the best fit, we considered that their belief in period *k* after no lie was observed was given by the empirical proportion of lie in period *k* obtained from the overall population of senders -humans and machines- when no lie was observed up to period $k-1$.

Since the beliefs are almost identical for rational and coarse receivers after having observed the first false message, we focus on receivers’ choices conditional on no lie being observed so far.^40^^,^^41^

We first test whether the data are best explained assuming all receivers are Coarse or assuming all receivers are rational. We obtain that data are best explained when all receivers are coarse (the log-likelihood ratio is higher than 60 and extremely significant).

We next move to estimating a mixed model in which we allow receivers to be either coarse or rational and we estimate the proportion of the two types (as well as the noise parameter $\lambda$). Formally, let

$$\begin{aligned} LR=\prod _{i=1}^{n}\prod _{s\in S_{d,a}^{i}}\prod _{k=1}^{5}[x_{is}^{r}\exp \lambda V_{k}(b_{ks}^{r},d_{ks}^{i})+(1-x_{is}^{r})\exp \lambda V_{k}(b_{ks}^{c},d_{ks}^{i})] \end{aligned}$$

where $S_{d,a}^{i}$ is the set of sessions in which receiver *i* is matched either with a deceiving sender or an automaton, $x_{is}^{r}$ is a binary variable equal to 1 if the receiver is categorized as rational and equal to 1 if he is categorized as coarse in session *s*, and $d_{ks}^{i}$ is the decision of receiver *i* in period *k* of session *s*.

Maximizing LR with respect to $$x_{is}$$ yields $$x_{is}=1$$ if $$ d^{i}_{s}$$ is better explained (higher likelihood ratio) by referring to rational belief, $$b^{r}$$, than coarse belief, $$b^{c}$$, and 0 otherwise.

In standard sessions, maximizing *LR* with respect to $(x_{is})$ and $\lambda$, this two population model gives a much higher likelihood ratio and a much higher $\lambda$ than models with only one (homogeneous) population. Our estimation gave $\lambda =3.5$ and a 58% share of coarse receivers.

We also considered adding a third type of receiver referred as skeptical who would consistently use $b^{s}=0.5$. In this case, we found that the share of coarse was 55%, the share of rational was 30% and the share of skeptical was 15% with $\lambda =4.1.$ These results are quite in line with our categorization in terms of $\backslash$-receivers and V-receivers. Besides, trying to connect the two methods, we found that 77% of $\backslash$-receivers were categorized as coarse receivers ($x_{is}=0$) and 90% of V-receivers were categorized as rational ($x_{is}=1$) according to the statistical method, thereby giving some extra support as to why our heuristic categorization captures an essential element that differentiates the behaviors of coarse receivers from those of rational receivers.

We obtain qualitatively equivalent results with the other variants of the game (except 5 period sessions), with a fraction of coarse receivers varying between 51% and 58% in the main ones.

In the 5 period sessions, in accordance with our difficulties in identifying receivers’ behaviors, we obtain a low value for $\lambda$, 0.63.

## Footnotes

1 For more details on the Red Orchestra, see Trepper (1975) or Perrault (1967).

2 In a very different context, one can also mention the effect of employment protection on worker effort for which Ichino and Ripahn (2005) observe that there is much less absenteism (the analog of cheating/lying) during probation periods than after (where the time of the hiring decision plays the role of the high stake event).

3 Not informing players of their opponents preferences has precedents in the experimental literature, see in particular Huck et al. (2011) or Esponda and Vespa (2018). While this is non-standard, it has the advantage of better fitting the informational conditions of a number of applications in which to assess others’ preferences one has to rely on the observation of their behaviors (this revealed preference view agrees with most of the motivating examples discussed above with the exception of the Red Orchestra, as in this case it was hard for the Russians to ignore that the Germans had preferences opposed to them).

4 When rational, a subject would play optimally as in standard equilibrium approaches. When coarse, a sender would randomize between telling a lie and telling the truth in every period because she would fail to see the impact of her current lie on future behavior.

5 In the chain-store game, the monopolist would like to be considered to be always fighting in case of entry.

6 Sometimes, references to homemade (i.e subjective) beliefs were required (Camerer and Weigelt 1988), or some departures from the theoretical predictions were observed close to the end of the interaction in the mixed strategy phase (Jung et al. 1994).

7 Gneezy (2005) is also suggestive of the presence of lying costs. But, the aversion to lying has been questioned in recent papers which have considered related but different environments (see Embrey et al. (2015) or Vespa and Wilson (2016, 2020)).

8 Observe that we do not define benevolent senders as having the same preferences as receivers but being otherwise free to send whatever message they like. We force benevolent senders to transmit truthful messages.

9 We refer to such patterns of behavior as deceptive tactic as we believe they capture common sense of deception insofar as they contain a good looking phase (up to the key period) followed by an exploitation phase (at the key period).

10 In our experiment (and we believe in a number of applications too), subjects had explicitly access to such aggregate statistics.

11 The reason why rational senders do so follows an equilibrium logic. Rational senders find it optimal to lie by the key period. After this period, they are indifferent between lying and telling the truth since both type of receivers choose $d=\frac{1}{2}$. The average 10 false and 10 truthful messages is necessary for receivers to choose $d=\frac{1}{2}$. And if *d*s were different from $\frac{1}{2}$, senders would either choose to send false messages with probability 0 or 1.

12 This is so for any $\beta$ in [0.2, 0.57]. Computations needed to find out the values of the bounds of this interval are available upon request.

13 The effect on $\widehat{d}$ is however small. These vectors restricted to the first five periods are (0.45, 0.41, 0.346, 0.25, 0.18) for $\alpha =0.1$ and (0.4, 0.33, 0.25, 0.17, 0.1) for $\alpha =0.2$.

14 The difference between the payoff obtained over the first five periods with the deceptive tactic and a tactic in which the sender lies in the first period and randomizes afterwards drops by 27% compared to the 20 period version. Note that if we were to assume that senders play noisy best-responses as in the Quantal response equilibrium, this drop of payoff would imply that the deceptive tactic is slightly less used in the 5 period version.

15 It seems that the unpredictability worked well since we did not observe that receivers derived significantly different payoffs in the last period compared to the previous ones ($p>0.85$, paired T-test)

16 The instructions mentioned: “Toutes les périodes d’une session n’ont pas la même importance. On associe un poids à chaque période. Toutes les périodes ont un poids de 1 sauf une qui a un poids de 5, la période 5.” (All periods do not correspond to the same stake. All periods have weight 1 except period 5 which has weight 5. English translation.) Given that receivers were not told senders’ payoffs, this formulation in terms of stake (or “importance” in french) is the most suggestive that the weights are common to both parties.

17 In line with the literature on ambiguity, one could argue that when agents ignore others’ preferences they adopt the worst-case theory, which would lead receivers to have the correct view that human senders are malevolent in this case.

18 Besides, in a number of contexts, past behaviors are often framed in the shape of summary statistics somewhat similar to the aggregate lie rate that we consider in our experiment.

19 Since the game was shorter, participants played it 10 times rather 5 times.

20 The frequency 0.28 is significantly different from 0.14 ($p<10^{-4}$, $n=400$, T-test) .

21 In order to obtain more data, we bundled for this statistic, the payoffs obtained by human senders following a deceptive tactic and the payoffs that the automata senders would have obtained if they had sent a false message in period 5, supposing that $d_{5}$ would have remained the same in that case. Let us also mention that the difference between the average payoffs in the two groups is negligible, 362 with automata and 360 with human senders.

22 We ran the test for the baseline treatment but not for the variants. With 3 or 4 sessions, we did not have enough data to do so.

23 This is because rational senders would pick the deceptive tactic and among the $1-\beta$ coarse senders, $\frac{1}{ 2^{5}}=\frac{1}{32}$ of them would also behave (by chance) according to the deceptive tactic.

24 Proposition 2 predicts that the deceptive tactic provides a payoff of 371, close to the 361 we observe, and that the revenue if the first false message appears before period 5 is 235. The 292 payoff we observe can be explained by the high variance of the *d*s after a false message. While best-response would lead receivers to choose $d=0.5$ in such events, we observe more variations, which may be attributed to the desire of receivers knowing they face malevolent senders to guess what the right state is as kids would do in rock-paper-scissor games. Such a deviation from best-response on the receiver side is beneficial to senders.

25 For other histories, there is no difference in the behaviors of rational and coarse receivers in the ABSE shown in Proposition 2.

26 In fact, because receivers’ decisions are somehow noisy, we allow $d_{k+1}$ to be higher than $d_{k}$ by at most 0.1, not more than once and for $k<4$.

27 It is difficult to distinguish the ABSE rational receivers’ behavior on the graph since rational receivers behave like coarse receivers in the first four periods (so as not to be detected by rational senders) and V-receivers behave almost exactly like ABSE rational receivers in periods 4 and 5.

28 We also ran 2 other variants with 132 participants that we will briefly discuss. Free sessions (4 sessions). This treatment was identical to the baseline treatment except that human senders were not constrained to send a specified number of truthful or false messages. We observe that the average ratio of false messages communicated by human senders is equal to 0.46, close to 0.5. We do not observe a strong bias toward truth-telling. The observed frequency of deceptive behavior is 0.28 and the observed ratio of V-receivers is $24\%$ . Both frequencies are extremely close to those obtained in the baseline treatment. Belief sessions (4 sessions). On the top of the interactions, receivers were asked, in each period, to report their belief regarding the probability with which they were facing a machine or a human sender. We observe that this extra query did not affect the aggregate behaviors of the players in terms of the lying strategy or the sequence of $d_{k}$. We analyze the extent to which the two populations of receivers also differ in their belief regarding whether they are matched with a machine or a human sender. As it turns out, we observe major differences. $\backslash$-receivers do not pay attention specifically to period 5. For them, a truthful message in period 4 is a good indicator that the sender is less likely to be a human sender (although both in the experimental data and in the ABSE with $\beta =\frac{1}{4}$, malevolent senders seldom send a first false message in period 4). $\backslash$-receivers do not perceive as more informative a truthful message sent in period 5 than a truthful message sent in period 4. These observations are consistent with our interpretation of $\backslash$-receivers as reasoning in terms of coarse analogy classes. Conditional on not observing any false message, V-receivers’ belief about the probability of being matched with an automaton is almost not modified by a truthful message in period 4 while it increases heavily after a truthful message in period 5. These observations are in agreement with the interpretation that V-receivers do not expect human senders to send their first false message in period 4 but perceive that a human sender if she has not sent her first false message prior to period 5 is very likely to do so in period 5. These elements are qualitatively reminiscent of the equilibrium strategy of the rational receiver in the ABSE.

29 The SE predicts that, conditional on not having sent a false message before, malevolent senders send a false message with a probability close to 0.5 in the first 4 periods, except in period 4 in which this probability is close to 0.2. Besides the probability to send a false message in period 5 conditional on not having sent any before is 1. With the randomizing 50:50 strategy, by definition, senders send a false with a probability 0.5 in every period conditional on not having sent one before. Therefore, in order to distinguish the two, we need to focus on periods 4 and 5. In period 4, among the 46 senders who did not send a false message in a prior period, 46% send a false message and in period 5, among the 25 senders who did not send a false message in a prior period, 48% send a false message. These two observations are much more in line with the 50:50 behaviors.
