# Supplementary material for: An experiment on deception, reputation and trust
Source: Exp Econ. 2020 Dec 1;24(3):821–53. doi: 10.1007/s10683-020-09681-9 (PMC8550209; doi:10.1007/s10683-020-09681-9)
Supplement: Supplementary file 1 — Supplementary material 1 (pdf 128 KB) [file 10683_2020_9681_MOESM1_ESM.pdf]

6.3 Experimental Instructions
FOR ONLINE PUBLICATION

Original Senders Instructions

Vous allez participer à une expérience économique. Suivant les décisions que vous allez prendre, vous pourrez obtenir une certaine somme d'argent que nous vous paierons à la fin de cette expérience.

Cette expérience prendra la forme de cinq sessions identiques auxquelles vous participerez les unes après les autres. Les participants à l'expérience auront deux rôles différents : experts ou décideurs. Vous tiendrez le rôle d'expert et les participants qui nous rejoindront à venir seront des décideurs. Chaque expert sera associé, pour chacune des sessions, à un décideur.

Chacune des sessions est composée de 20 périodes. Lors de chacune de ces 20 périodes, votre gain dépendra du côté sur lequel est tombée une pièce (un côté indiquant 0 et l'autre indiquant 1) et du choix effectué par le décideur auquel vous serez associé pour la durée d'une session (vous êtes associés à un même décideur pour les 20 périodes d'une session mais changez de décideur associé à chaque session). Votre gain au cours d'une période sera d'autant plus élevé que le choix du décideur, une valeur entre 0 et 1, sera éloigné de la valeur obtenue lors du tirage (0 ou 1).

Chaque période se déroule de la façon suivante. L'ordinateur jette une pièce virtuelle. Un côté de cette pièce indique 0 et l'autre 1. Cette pièce n'est pas truquée si bien que, avec une probabilité ½, elle tombe du côté 1 et, avec une probabilité ½, elle tombe du côté 0. De plus, les tirages de la pièce pour chacune des périodes sont indépendants. En tant qu'expert, vous êtes informé du côté sur lequel la pièce est tombée alors que le décideur auquel vous êtes associé ne l'est pas.
Après avoir été informé du côté sur lequel la pièce est tombée, vous devez choisir d'envoyer l'un des deux messages suivants au décideur auquel vous êtes associé : « La pièce est tombée du côté 0 » ou « La pièce est tombée du côté 1». Vous pouvez donc choisir d'envoyer une information exacte ou une information inexacte. Le système est ainsi fait que lors de chaque session, vous serez contraint d'envoyer une information exacte 9, 10 ou 11 fois sur les 20 périodes et, lors des autres périodes, des informations inexactes. L'ordinateur vous indiquera en début de session combien de fois vous devrez envoyer une information exacte pour la session en cours.

Le déroulement des opérations lors d'une période est le suivant :

1. L'ordinateur procède au tirage de la pièce. Vous, expert, êtes informé du côté sur lequel la pièce est tombée mais pas le décideur auquel vous êtes associé.
2. Vous faites le choix d'un message à envoyer au décideur : « la pièce est tombée du côté 0 » ou « la pièce est tombée du côté 1». Sur l'écran apparaîtra en vert le choix correspondant à une information exacte et en rouge celui correspondant à une information inexacte. De plus, sur cet écran, vous serez informé du nombre de messages exacts et de messages inexacts qu'il vous reste à envoyer.
3. Le décideur auquel vous êtes associé prend une décision. Cette décision correspond au choix d'une valeur entre 0 et 1.
4. Le décideur est informé du côté sur lequel la pièce est effectivement tombée (et découvre si vous avez envoyé une information exacte ou inexacte lors de cette période).
5. L'ordinateur calcule les gains de la période et la période suivante peut commencer (sauf lors de la période 20 où il faut attendre que toutes les paires expert/décideur aient fini leurs 20 périodes pour pouvoir entamer la session suivante).

Afin de mieux anticiper leurs décisions et réactions, voici quelques éléments sur les décideurs auxquels vous pouvez être associés ainsi que sur vos gains

Le calcul des gains en jetons d'un décideur s'effectue de façon à ce que, à chaque période, plus sa décision se rapproche du côté sur lequel la pièce est tombée plus son gain est élevé. Les gains en jetons seront transformés en gains en euros selon des règles différentes pour les experts et les décideurs (CF ci-après).
A chaque période, la somme de votre gain en jetons et de celui du décideur auquel vous êtes associé est égale à 100.
Ainsi, plus la décision prise par le décideur sera proche de la valeur du côté sur lequel est tombée la pièce, plus son gain en jetons sera élevé et plus le vôtre sera faible. Réciproquement, plus la décision prise par le décideur sera éloignée de la valeur du côté sur lequel est tombée la pièce, plus son gain en jetons sera faible et plus le vôtre sera élevé.
Plus précisément, le calcul des gains en jetons lors d'une période se fait de la façon suivante. Supposons que la valeur de la décision du décideur auquel vous êtes associé soit «D».
Si la pièce est tombée du côté 0, le gain du décideur est égal à : $100 \times (1-D^2)$ et votre gain est égal à : $100 - 100 \times (1-D^2)$. Exemple : la pièce est tombée du côté 0 et le décideur a choisi : 0,4 ; son gain est alors égal à 84 et le vôtre à 16.
Si la pièce est tombée du côté 1, le gain du décideur est égal à : $100 \times (1-(1-D)^2)$ et votre gain est égal à : $100 - 100 \times (1-(1-D)^2)$. Exemple : la pièce est tombée du côté 1 et le décideur a choisi : 0,3 ; son gain est alors égal à 51 et le vôtre à 49.

Les décideurs connaissent la valeur de leurs gains en fonction de leur décision et du côté sur lequel la pièce est tombée. En revanche, ils ne savent pas comment vos gains (en tant qu'experts) sont calculés.

Enfin, la formation des couples expert/décideur se fait de la façon suivante. Lors de chaque session, chaque décideur est associé avec une probabilité 4/5 avec un expert de votre type, « humain », qui envoie une information inexacte en moyenne 10 fois sur les 20 périodes d'une session, (sans connaître la forme des gains d'un tel expert) ou, avec une probabilité 1/5, avec un expert de type « machine » (un programme d'ordinateur) qui lui donnera une information exacte sur le côté sur lequel la pièce est tombée à chaque période. Les décideurs connaissent ces 2 possibilités d'association et les 2 probabilités (4/5 et 1/5).
Les experts de type « machine » envoyant toujours une information exacte, à la fin de la première période où vous aurez envoyé une information inexacte, lorsque l'ordinateur informera le décideur que vous avez envoyé une information inexacte (à la fin de la période en question) cela révélera que vous êtes un expert de votre type (envoyant 10 fois sur 20, en moyenne, un message inexact) et non pas un expert « machine » disant toujours la vérité.
Nota : la formation des couples à chaque période se fait de façon anonyme et aléatoire. Ni vous ni les décideurs ne sauront jamais avec qui vous avez été associés. A chaque nouvelle session, on procède à une nouvelle formation des couples expert/décideur.

Derniers éléments :
Votre gain à chaque période est comptabilisé en une unité de compte que nous appellerons le *jeton*. A la fin de l'expérience, la somme des jetons que vous aurez gagné au cours de l'ensemble des périodes sera calculée par l'ordinateur et votre gain en jetons sera converti en un gain en Euros, avec pour taux de change : un jeton est égal à 0,45 centimes. Nota : la valeur du jeton pour un décideur est 0,15 centimes, ceci afin que les paiements que vous recevrez, décideurs et experts, soient du même ordre.

Toutes les périodes d'une session n'ont pas la même importance. On associe un *poids* à chaque période. Toutes les périodes ont un poids de 1 sauf une qui a un poids de 5, la période 5. Le gain au cours d'une période est multiplié par le poids de cette période pour déterminer le nombre de jetons gagnés lors de cette période. Cette période est donc 5 fois plus importante que les autres du point de vue des gains.
Par ailleurs, le tableau en bas de l'écran affichera, au cours des sessions, le poids de chaque période, les messages que vous avez déjà envoyés (en indiquant s'ils sont exacts ou non) et les décisions prises par le décideur auquel vous êtes associé ainsi que vos gains en jetons obtenus lors des périodes précédentes.

Original Receivers Instructions

Vous allez participer à une expérience économique. Suivant les décisions que vous allez prendre, vous pourrez obtenir une certaine somme d'argent que nous vous paierons à la fin de cette expérience.

Cette expérience prendra la forme de cinq sessions identiques auxquelles vous participerez les unes après les autres.

Chacune de ces sessions est composée de 20 périodes. Lors de chacune de ces 20 périodes, votre gain dépendra du côté sur lequel est tombée une pièce (un côté indiquant 0 et l'autre indiquant 1) et de votre décision. Votre gain au cours d'une période sera d'autant plus élevé que votre décision, D, sera proche de la valeur obtenue lors du tirage (0 ou 1). Notons que le poids de chacune des périodes n'est pas le même (CF description ultérieure).

Au moment où vous devrez prendre votre décision vous ne serez pas informés du côté sur lequel la pièce est tombée. Néanmoins, vous disposerez d'un certain nombre d'informations. Le tirage se déroule de la façon suivante. Lors de chacune des périodes, l'ordinateur jette une pièce virtuelle. Un côté de cette pièce indique 0 et l'autre 1. Cette pièce n'est pas truquée si bien que, avec une probabilité ½, elle tombe du côté 1 et, avec une probabilité ½, elle tombe du côté 0. Les tirages de la pièce pour chacune des périodes sont indépendants (le fait que lors d'une période, la pièce tombe du côté 1 ne rend pas plus probable qu'elle tombe du côté 0 à la période suivante).
Afin de vous aiguiller, vous êtes associés lors de chacune des sessions de 20 périodes à un "expert" (vous resterez avec le même expert pendant les 20 périodes d'une session mais changerez d'expert lors de chacune des cinq sessions). Un expert est toujours parfaitement informé de la valeur du tirage à chaque période. Au début de chaque période, avant que vous ne preniez votre décision, votre expert vous enverra l'un des deux messages suivants : « La pièce est tombée du côté 0 » ou « La pièce est tombée du côté 1».
Il existe deux types d'experts, les experts de type A et les experts de type B. Un expert de type A est un programme informatique qui a été programmé de façon à toujours envoyer une information exacte. Un expert de type B est un individu, en moyenne, il envoie une information exacte la moitié du temps (il y est contraint). C'est-à-dire qu'il communique la valeur exacte du tirage 10 fois sur 20 et une valeur incorrecte, les 10 autres fois (il s'agit d'une moyenne et il n'est pas certain que sur les 20 périodes, un expert de type B mente exactement 10 fois).
Au début de chaque session, avec une probabilité 1/5, vous êtes associés avec un expert de

type A et avec une probabilité 4/5, vous êtes associés avec un expert de type B. Vous ne savez pas quel est le type de votre expert.

Le déroulement des opérations lors d'une période est donc le suivant :

1. L'ordinateur procède au tirage de la pièce. Vous ne savez pas de quel côté la pièce est tombée mais l'expert lui est informé du côté sur lequel est tombée la pièce.
2. Votre expert vous envoie un message : « la pièce est tombée du côté 0 » ou « la pièce est tombée du côté 1».
3. Vous devez prendre une décision. Cette décision correspond au choix d'une valeur entre 0 et 1, bornes incluses (NB : vous n'êtes pas restreints à prendre une décision extrême, D = 0 ou D = 1, et pouvez prendre une décision intermédiaire pour mieux tenir compte de votre incertitude sur le tirage).
4. La machine vous indique de quel côté la pièce est effectivement tombée.
5. L'ordinateur calcule votre gain pour la période.

Le calcul du gain d'une période se fait de la façon suivante. Supposons que la valeur de votre décision soit «D». Si la pièce est tombée du côté 0, votre gain est égal à :

$$100 \times (1- D^2)$$

Si la pièce est tombée du côté 1, votre gain est égal à :

$$100 \times (1- (1-D)^2)$$

Il apparaît nettement que plus D est proche de la valeur sur laquelle la pièce est tombée, plus votre gain est élevé. Ainsi, si vous avez pris pour décision 0,6 et que la pièce est tombée du côté 1, votre gain sera égal à :

$$100 (1-(0,4)^2) = 84$$

Et, si vous avez pris pour décision 0,4 et que la pièce est tombée du côté 1, votre gain sera égal à :

$$100 (1-(0,6)^2) = 64$$

Revenons sur la méthode que nous utilisons pour calculer vos gains à chaque période. Celle-ci a été choisie de façon à ce que vous maximisiez votre espérance de gain pour la période en

question en choisissant un D correspondant à votre croyance sur la valeur espérée du tirage. Si vous pensez que la pièce est tombée avec une probabilité 0,6 sur le côté 1 et avec une probabilité 0,4 sur le côté, votre croyance sur la valeur espérée du tirage est 0,6x1 + 0,4x0 = 0,6. 0,6 est alors la meilleure décision possible si vous avez effectivement cette croyance. (Bien que vous pensiez, dans ce cas, que la pièce a plus de chances d'indiquer le côté 1, vous n'avez pas intérêt à prendre comme décision : 1. En effet, une telle décision vous apporterait un gain espéré de 60 alors que prendre pour décision 0,6, vous apporterait un gain espéré de 76).

Votre gain à chaque période est comptabilisé en une unité de compte que nous appellerons le *jeton*. A la fin de l'expérience, la somme des jetons que vous aurez gagné au cours de l'ensemble des périodes sera calculée par l'ordinateur et votre gain en jetons sera converti en un gain en Euros, avec pour taux de change : un jeton est égal à 0,15 centimes.

Derniers éléments :
A la fin de chaque période, après avoir pris votre décision, vous êtes informés de la véritable valeur du tirage pour cette période. Vous pouvez ainsi découvrir si votre expert a menti ou dit la vérité lors de cette période.
Toutes les périodes d'une session n'ont pas la même importance. On associe un *poids* à chaque période. Toutes les périodes ont un poids de 1 sauf une qui a un poids de 5, la période 5. Le tableau en bas de l'écran vous rappellera, tout le long de la session, le poids de chaque période. Le gain au cours d'une période est multiplié par le poids de cette période pour déterminer le nombre de jetons gagnés lors de cette période.

Instructions – Senders (translation)

You are going to take part in an economic experiment. Depending on the decisions you make, you will be able to receive a certain sum of money which we will pay you at the end of this experiment.

This experiment will be conducted over five sessions which you will take part in one after the other. The participants in the experiment will have two different roles: experts or decision-makers. You will be taking the role of expert and the participants joining us will be decision-makers. In each of the sessions, each expert will be partnered with a decision-maker.

Each session is composed of 20 periods. During each of these 20 periods, your earnings will depend on which side a coin has fallen (0 on one side and 1 on the other) and on the choice made by the decision-maker you are matched with throughout the session (you are matched with the same decision-maker for the 20 periods of a session, but change your matched decision-maker every session). The further away the decision-maker's choice, a value between 0 and 1, is from the value obtained in the throw (0 or 1), the higher your earnings during a period.

Each period will be conducted as follows. The computer throws a virtual coin. On one side of this coin is a 0 and on the other a 1. This coin is not fixed - it has a ½ probability of falling 1 side up and a ½ probability of falling 0 side up. Furthermore, in each session the coin is thrown independently. As the expert, you are notified which side the coin has fallen on, whereas the decision-maker you are partnered with is not.
After you have been notified which side the coin has fallen on, you must choose which of the following messages you are going to send to the decision-maker you are matched with: "The coin has fallen 0 side up" or "The coin has fallen 1 side up". So you can choose to send information which is either correct or incorrect. The system has been designed so that during each session, you will have to send correct information 9, 10 or 11 times throughout the 20 periods and, during the other periods, incorrect information. The computer will show you at the beginning of the session how many times you must send correct information for the current session.

The following procedure is carried out during a period:

1. The computer throws the coin. You, the expert, are notified which side the coin has fallen on, but the decision-maker you are matched with is not.

2. You choose a message to send to the decision-maker: "The coin has fallen 0 side up" or "The coin has fallen 1 side up". The correct information choice will appear on the screen in green and the incorrect information choice will appear in red. This screen will also show you the number of correct messages and incorrect messages you have left to send.
3. The decision-maker you are matched with makes a decision. This decision is the choice of a value between 0 and 1.
4. The decision-maker is notified which side up the coin has actually fallen (and finds out if you sent correct or incorrect information during this period).
5. The computer calculates the earnings for that period and the following period can begin (except during period 20 where it is necessary to wait until all the expert/decision-maker pairs have finished their 20 periods to be able to start the next session).

In order to better anticipate their decisions and reactions, here is some information about the decision-makers you may be matched with, and your earnings, too.

A decision-maker's earnings in tokens are calculated in such a way that, at the end of each period, the closer his decision is to the side the coin has fallen on, the higher his earnings. The earnings in tokens will be changed into earnings in Euros according to different rules for the experts and the decision-makers (Cf. below).
In each period, the amount of your earnings in tokens and that of the decision-maker you are matched with is equal to 100.
So the closer the decision-maker's decision is to the value of the side the coin has fallen on, the higher his earnings in tokens and the lower your earnings. Vice versa, the further away the decision-maker's decision is to the value of the side the coin has fallen on, the lower his earnings in tokens and the higher your earnings.
To be more precise, the earnings during a period are calculated as follows. Let's suppose that the value of the decision made by the decision-maker you are matched with is "D".
If the coin falls 0 side up, the decision-maker's earnings are equal to: $100 \times (1-D^2)$ and your earnings are equal to: $100 - 100 \times (1-D^2)$. Example: the coin falls 0 side up and the decision-maker chose: 0.4; his earnings are then equal to 84 and yours are 16.
If the coin falls 1 side up, the decision-maker's earnings are equal to: $100 \times (1-(1-D)^2)$ and your earnings are equal to: $100 - 100 \times (1-(1-D)^2)$. Example: the coin falls 1 side up and the decision-maker chose: 0.3; his earnings are then equal to 51 and yours are 49.
The decision-makers know the value of their earnings according to their decision and the side on which the coin fell. However, they do not know how your earnings (as experts) are calculated.

Finally, the expert/decision-maker pairings are formed as follows. During each session, there is a 4/5 probability of every decision-maker being matched with your type of expert, "human", who sends incorrect information on average 10 times over the 20 periods in a session, (without knowing the form of such an expert's earnings) or a 1/5 probability of being matched with a "machine" type of expert (a computer program) which will give him correct information about the side the coin fell on each period. The decision-makers know about these 2 partnership possibilities and the 2 probabilities (4/5 and 1/5).
As the "machine' type of expert always sends correct information, at the end of the first period during which you have sent incorrect information, when the computer notifies the decision-maker that you have sent incorrect information (at the end of the period in question), it will reveal that you are your type of expert (sending an incorrect message on average 10 times out of 20) and not a "machine" expert always telling the truth.
NB: the match for each period are anonymously and randomly formed. Neither you nor the decision-makers will ever know who you have been partnered with. At each new session, a new expert/decision-maker pairing is formed.

Final information:
Your earnings for each period are counted in units of account we will call *tokens*. At the end of the experiment, the amount of tokens you have won during all the periods will be calculated by the computer and your earnings in tokens will be converted into earnings in Euros, with an exchange rate of one token being equal to 0.45 centimes. NB: the value of the token for a decision-maker is 0.15 cents, so that the payments both decision-makers and experts receive are of the same order.

Not all the periods in a session have the same importance. A *weighting* is given to each period. All the periods have a weighting of 1 except one which has a weighting of 5 - Period 5. The earnings during a period are multiplied by the weighting of this period to determine the number of tokens won during this period. This period is therefore 5 times more important than the others from an earnings standpoint.
Furthermore, during the sessions, the table at the bottom of the screen will display the weighting of every period, the messages that you have already sent (indicating if they are correct or not) and the decisions taken by the decision-maker you are matched with as well as the earnings you have received during the previous periods.

Instructions – Receivers (Translation)

You are going to take part in an economic experiment. Depending on the decisions you make, you will be able to receive a certain sum of money which we will pay you at the end of this experiment.

This experiment will be conducted over five sessions which you will take part in one after the other.

Each of these sessions is composed of 20 periods. During each of these 20 periods, your earnings will depend on the side on which a coin has fallen (0 on one side and 1 on the other) and on your decision. Your earnings during a period will be higher the closer your decision, D, is from the value obtained in the throw (0 or 1). Please note that the weighting of each of the periods is not the same (Cf. description below).

At the time you have to make your decision, you will not have been notified which side the coin has fallen on. Nevertheless, you will have a certain amount of information. The throws will take place as follows. During each of the periods, the computer throws a virtual coin. On one side of this coin is a 0 and on the other a 1. This coin is not fixed - it has a ½ probability of falling 1 side up and a ½ probability of falling 0 side up. The throws of the coin for each of the periods are independent (meaning that during a period, if the coin falls 1 side up, it does not make it more likely that it will fall 0 side up in the next period).
In order to direct you, you are matched during each of the 20-period sessions by an "expert" (you will stay with the same expert during the 20 periods of a session but will change expert for each of the five sessions). An expert is always perfectly informed of the value of each period's throw. At the beginning of each period, before you make your decision, your expert will send you one of the two following messages: "The coin has fallen 0 side up" or "The coin has fallen 1 side up".
There are two types of experts, Type A experts and Type B experts. A Type A expert is a computer program which has been programmed to always send you correct information. A Type B expert is an individual person and on average he sends correct information half of the time (he is obliged to do so). That means that he communicates the correct value of the throw 10 times out of 20, and an incorrect value the other 10 times (it is an average and it is not certain that over the 20 periods, a Type B expert lies exactly 10 times).
At the beginning of each session, there is a 1/5 probability of you being matched with a Type A expert and a 4/5 probability of you being matched with a Type B expert. You do not know which type of expert you have.

The following procedure is carried out during a period:

1. The computer throws the coin. You do not know which side the coin has fallen on, but the expert is informed which side the coin has fallen on.
2. Your expert sends you a message: "The coin has fallen 0 side up" or "The coin has fallen 1 side up".
3. You must make a decision. This decision involves the choice of a value between 0 and 1 inclusive (NB: you are not restricted to making an extreme decision, D = 0 or D = 1, and you can make a decision in between the two to better reflect your uncertainty about the throw).
4. The machine tells you on which side the coin actually fell.
5. The computer calculates your earnings for the period.

The earnings during a period are calculated as follows. Let's suppose that the value of your decision is "D". If the coin falls 0 side up, your earnings are equal to:

$$100 \text{ x } (1- D^2)$$

If the coin falls 1 side up, your earnings are equal to:

$$100 \text{ x } (1- (1-D)^2)$$

Clearly, the closer D is to the value on which the coin has fallen, the higher your earnings. So if you decided on 0.6 and the coin fell 1 side up, your earnings will be equal to:

$$100 \ (1-(0.4)^2) = 84$$

And if you decided on 0.4 and the coin fell 1 side up, your earnings will be equal to:

$$100 \ (1-(0.6)^2) = 64$$

Let's go back to the method we use to calculate your earnings for every period. It has been chosen so that you maximise the earnings to be had for the period in question by choosing a D reflecting what you believe the expected value of the throw to be. If you think that the coin has fallen with a 0.6 probability of being 1 side up and with a 0.4 probability of being the other side up, your belief of the expected value of the throw is 0.6x1 + 0.4x0 = 0.6. 0.6 is therefore the best possible decision if this is what you actually believe. (Although you might have

thought, in this case, that the coin is more likely to be 1 side up, you would be well advised not to decide on 1. In fact, such a decision would bring you expected earnings of 60, while deciding on 0.6 would bring you expected earnings of 76).

Your earnings for each period are counted in units of account we will call *token*s. At the end of the experiment, the amount of tokens you have won during all the periods will be calculated by the computer and your earnings in tokens will be converted into earnings in Euros, with an exchange rate of one token being equal to 0.15 cents.

Final information:
At the end of each period, after making your decision, you are notified of the true value of the throw for this period. You can then find out whether your expert has lied or told the truth during this period.
Not all the periods in a session have the same importance. A *weighting* is given to each period. All the periods have a weighting of 1 except one which has a weighting of 5 - Period 5. The table at the bottom of the screen will display the weighting of every period throughout the session. The earnings during a period are multiplied by the weighting of this period to determine the number of tokens won during this period.
